# Alcohol-Specific Computerized Interventions to Alter Cognitive Biases: A Systematic Review of Effects on Experimental Tasks, Drinking Behavior, and Neuronal Activation

**DOI:** 10.3389/fpsyt.2019.00871

**Published:** 2020-01-09

**Authors:** Hallie M. Batschelet, Maria Stein, Raphaela M. Tschuemperlin, Leila M. Soravia, Franz Moggi

**Affiliations:** ^1^University Hospital of Psychiatry, Translational Research Center, Division of Clinical Research, University of Bern, Bern, Switzerland; ^2^Department of Clinical Psychology and Psychotherapy, University of Bern, Bern, Switzerland; ^3^Clinic Suedhang, Center for Treatment of Addictive Disorders, Kirchlindach, Switzerland

**Keywords:** alcohol use disorder, cognitive bias modification, approach bias, attentional bias, inhibition, alcohol-specific computerized intervention

## Abstract

**Background:** In patients with alcohol use disorder, novel interventions to increase abstinence have attracted growing attention. Interventions aimed at modifying cognitive biases linked to alcohol use [i.e. cognitive bias modification (CBM)] may serve as an add-on to standard therapy. This systematic review thoroughly aggregates existing data on the effects of three alcohol-specific computerized interventions, namely attentional bias modification (AtBM), approach bias modification (ApBM), and inhibition training (IT). In doing so, each CBM’s effects on experimental tasks assessing the relevant biases, drinking behavior, and neurophysiology are summarized. Also, the influence of drinking behavior severity and motivation to change drinking behavior are discussed.

**Methods:** A literature search was conducted in four databases for original research articles published between 2000 and May 2019. Studies were eligible if investigating the effects of alcohol-specific computerized interventions (AtBM, ApBM, IT) on drinking behavior, bias change, and/or neurophysiology. Forty eligible articles were classified as being either a non-clinical experimental lab study (ELS) or clinical randomized-controlled trial (RCT) and summarized.

**Results:** While AtBM seems to influence attentional bias, its effects on drinking behavior are inconsistent. As for ApBM, the best effects on drinking behavior are obtained in clinical samples. Effects of ApBM on approach bias are mixed. Interestingly, those clinical RCTs which investigated ApBM effects on bias change as well as on drinking outcome, reported consistent effects in both measures (i.e. either effects on bias *and* drinking or no effects). Studies on IT are limited to non-clinical samples and show inconsistent effects on drinking behavior. Considering ITs effects on implicit semantic associations, most studies do not support the conceptualization of IT as a form of memory bias modification, while reports on IT’s effects on inhibitory control are still incomplete. Conclusions about the overall influence of drinking behavior severity are hampered by the non-uniform use of sample descriptions.

**Conclusions:** In clinical samples, ApBM has shown more consistent beneficial effects, while evidence on AtBM is more inconsistent, and data on IT still lacks important information. Conclusions about the influence of drinking behavior severity would be facilitated by a uniform use of clearly defined sample descriptions.

## Introduction

Alcohol use disorder (AUD) is a chronic health problem with serious biological, psychological, and social consequences (1). Despite sophisticated psychological and pharmacological treatment, patients frequently relapse (2), requiring the search for novel interventions to improve treatment. While cognitive behavioral treatments predominantly target conscious information processing, accumulating evidence for the importance of subconscious mechanisms is reflected less in treatment programs (3).

Certain alterations in the cognitive processing of alcohol-specific cues have been identified as being relevant in AUD. While the automatized tendency to process, memorize, and approach alcohol-related cues (4, 5) is pronounced in AUD patients, inhibitory processes to withhold from drinking alcohol are deficient (6). This behavioral research on cognitive processes in AUD is complemented by recent neuroscientific evidence for marked changes in brain activation while processing alcohol-related cues. Such changes are thought to play a pivotal role in the development and maintenance of AUD (7, 8). The automatic activation of both saliency and motivation-related networks is sensitized to alcohol-related cues, activation in brain areas linked to memory, interoception, and stress reactivity are altered, and the ability of brain regions involved in executive control to effectively exert that control function is impaired (9). Thus, both behavioral and neuroscientific evidence has highlighted the imbalance between automatized processes pushing patients toward alcohol-related cues and opposing control processes. Three specific substance-related maladaptive cognitive processes and the manipulation thereof have increasingly become the focus of research, namely biased selective attention (10) and approach tendencies (11) toward alcohol-related cues, as well as impaired inhibitory control (6).

The first of these processes, attentional bias (AtB), describes that alcohol-specific cues rapidly attract and subsequently hold selective attention (12). The extent of alcohol-specific AtB has been shown to be proportional both to alcohol consumption (13) and to predict future relapse rates in AUD (12). The second concept, approach bias (ApB) toward alcohol-specific stimuli, describes an automatic action tendency that biases a person to approach alcohol-related stimuli rather than avoid them. ApB has been shown to be pronounced in AUD, and to be related to the amount of alcohol consumption (14). Finally, inhibitory control is defined as the ability to suppress, delay, or change an inappropriate response (15). It represents a well-investigated component of impulsivity and executive functioning, which is deficient in individuals with AUD (6).

Motivated by cumulative evidence of altered cognitive processes in AUD, research has tested various interventions aimed at modifying attentional bias, approach tendencies, and inhibitory control impairments. The modification of such processes is commonly referred to as cognitive bias modification (CBM). Recently, there has been greater interest in alcohol-specific computerized interventions that target altering these deficits. On the one hand, the focus on alcohol-specific interventions is justified from a clinical perspective, since drinking behavior is often triggered by alcohol stimuli (5). On the other hand, the research perspective stresses the biased and misbalanced processing of such stimuli. The computerization of bias-altering interventions is not only cost-effective, but it also allows for repetitive training, an important prerequisite when automatized processing is to be targeted (16).

Several reviews and meta-analyses have covered literature on one or more of the three aforementioned alcohol-related CBMs, with some of them reaching a rather positive conclusion (17, 18), while others were more cautious (19–22). Most authors agreed that future studies should expand research to clinical samples, clarify which cognitive construct is best targeted, and enlarge knowledge about the moderating factors and mechanisms of action (17, 20, 23). In summarizing data on alcohol-related CBMs, it has been argued that it is necessary to disentangle experimental laboratory studies, which aim to investigate psychological mechanisms in non-clinical samples, from clinical trials, which test the efficacy in a clinical population (24). One important argument for this is the assumption that changing implicit processes will only translate into effective behavior change if participants are motivated to do so. Thus, motivation to change drinking behavior might be a prerequisite for CBM’s effects on drinking behavior. This argument is often heard in conjunction with the assumption that clinical populations are motivated to change their alcohol consumption, in contrast to heavily drinking students, for example. While this may be true when comparing means of both populations, the assumption that every AUD patient has a stable motivation to change alcohol consumption seems rather optimistic. Such an assumption is also challenged by research indicating that motivation in AUD patients varies over time and between subjects (e.g. 25).

This systematic review aims to extend and update those summaries as follows: First, the current database will be expanded by including the most recent research articles. Second, evidence will be thoroughly summarized separately for each of the computerized interventions targeting either attentional bias, approach bias, or inhibitory control processes. Conclusions are drawn for three outcomes, namely drinking behavior, experimental tasks, and neurophysiological effects, while distinguishing between non-clinical experimental lab studies and clinical randomized-controlled studies. In doing so, the potential role of motivation to change drinking behavior is acknowledged and systematically assessed. By visualizing whether an intervention affected the targeted bias and at the same time drinking behavior, light is shed on the assumption that drinking behavior change is associated with and driven by a bias change (see **Figures 2** and **3**).

Since the predictive value of non-clinical studies for potential benefits in a clinical sample is subject to question, we will compare the different studies’ effects while considering the investigated sample’s severity of drinking behavior. On the one hand, this will be a focus in the summary of each intervention. On the other hand, this review comprises an overall insight into the link between severity of drinking behavior and training effects across all three interventions.

## Methods

### Literature Search Strategy

**Figure 1:**
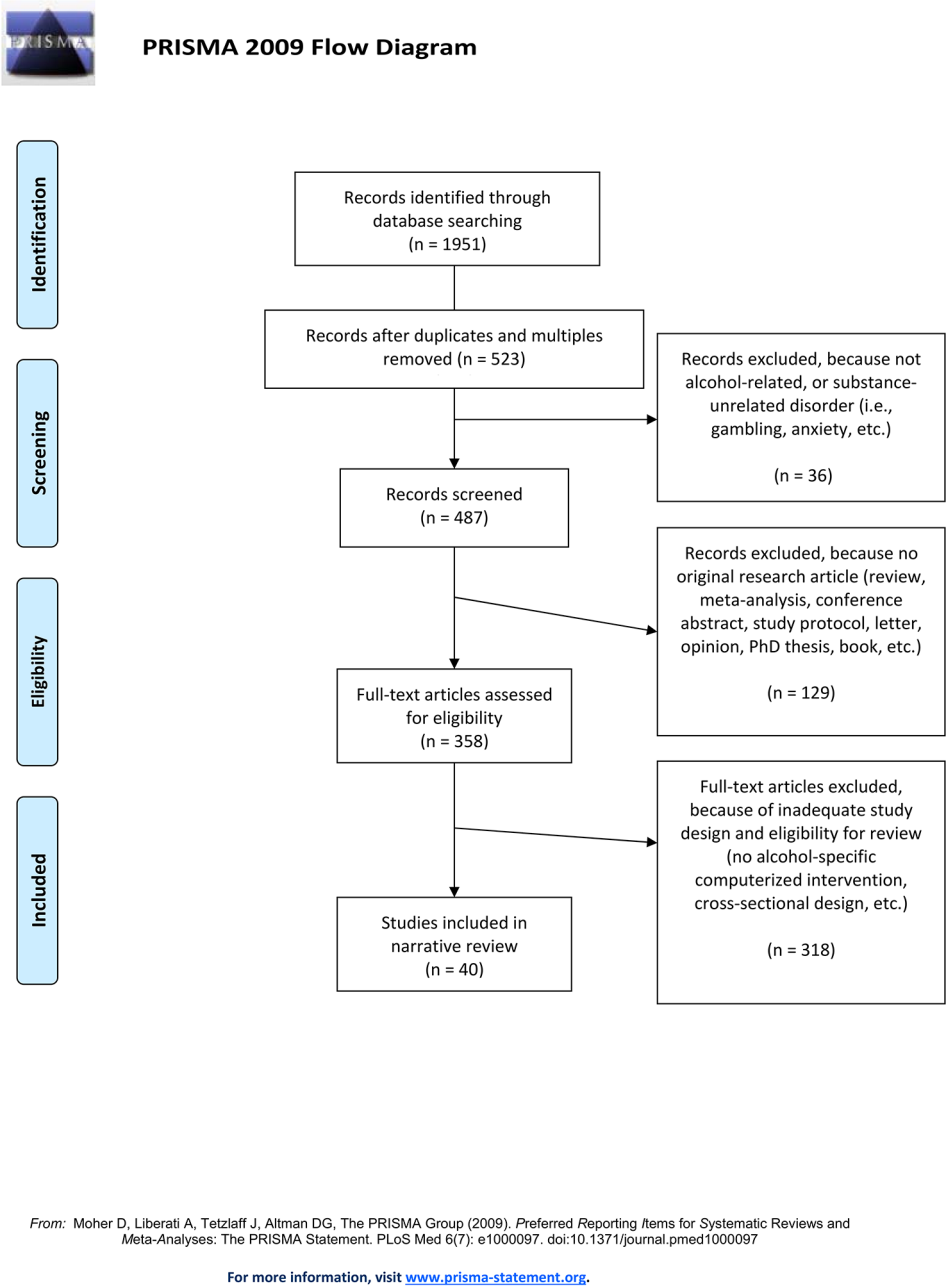
PRISMA flow chart of the conducted literature search.

Thorough literature searches in the databases PubMed Medline, Google Scholar, and PsycInfo were conducted in January and May 2019 to identify eligible publications papers on computerized alcohol-specific interventions for attentional and approach bias modification and inhibition training. The sample was to be alcohol-consuming, and studies were to include alcohol-specific interventions with the aim to alter the alcohol-specific biases (i.e. including alcohol-related stimuli). Both clinical and non-clinical samples were represented. The following keywords and all possible combinations thereof were entered into the search masks. Alcohol*, alcohol depend*, addict*, alcohol use disorder, harmful drinking, heavy alcohol use, heavy drink*, hazardous drink*, drink*, alcohol-specific computerized intervention, attention* bias, approach bias, approach-avoidance bias, cognitive bias modification, computerized training, inhibition, inhibitory control, and inhibition training. In order to identify additional papers, a search within Google Scholar was conducted for papers, in which already detected eligible papers for this review had been cited. Furthermore, the bibliographies of the included studies were reviewed. The literature search was restricted to original research articles including a computerized and alcohol-specific CBM intervention that had been published from 2000 through May 2019. **Figure 1** shows the selection process of literature included in this review, which ultimately included 40 eligible original research papers.

### Inclusion and Exclusion Criteria and Procedure

Eligible studies met the following criteria: the publications (a) were peer-reviewed and published quantitative research studies; (b) involved computerized interventions aimed to alter either attentional or approach bias or inhibitory control; c) implemented alcohol-related stimuli in the computerized intervention; and (d) examined either the effect of interventions on experimental and neurophysiological outcomes in a longitudinal design including a pre- and post-measurement, or the effect of the intervention on drinking outcomes at post-intervention only (including group comparisons of a post-intervention measure, such as taste test, relapse rates 1 year after intervention), and e) were written in English. Studies assessing bias, drinking, or neurophysiology cross-sectionally only were excluded.

Articles found in the literature search were assessed for eligibility by the first author (HB). Once all duplicates were removed, the remaining articles were screened for eligibility based on the inclusion and exclusion criteria. If inclusion was debatable, another author (MS) also assessed the paper and the case was discussed until an agreement was reached.

Studies were categorized as either experimental laboratory study (ELS) if they investigated non-clinical samples, or clinical randomized-controlled trials (RCT) if they described an RCT conducted in a clinical sample containing treatment-seeking patients with AUD. For all three CBMs, this review will initially summarize effects on experimental tasks, i.e. whether the CBM intervention affected the according bias. Thereafter, effects on drinking behavior are reported, as are neurophysiological effects, whenever applicable.

For all studies included in this review, the following data were extracted (see **Table 1**):

1. name of the first author(s) and publication year
2. the sample description (e.g. heavy drinkers, AUD patients), the sample’s mean age and standard deviation
3. the type of CBM intervention: attentional bias modification (AtBM), approach bias modification (ApBM), or inhibition training (IT)
4. the study design: non-clinical experimental lab study (ELS) or clinical randomized-controlled trial (RCT)
5. the different groups, to which participants were allocated (including size)
6. which task the intervention is based on (e.g. VPT, AAT, etc.)
7. the number of training sessions conducted
8. the outcomes, separated into experimental tasks, drinking behavior, and neuronal activity. As for experimental tasks, all assessed tasks were extracted. Drinking behavior outcomes were summarized according to how long after the intervention they were assessed.
9. remarks on important characteristic of study (e.g. no control group)

Table 1A–CDetailed overview of original research articles included in review according to CBM type [AtBM (1A), ApBM (1B), or IT (1C)], and study design/sample included [non-clinical (ELS) or clinical (RCT)]. Abbreviations are summarized below each sub-table.

**Table:** 

Table 1A: Attentional Bias Modification (AtBM) studies						
Reference	Sample/mean age (SD)	Experimental (EG) and control (CG) groups (n)	Intervention / # sessions	Outcomes		Remarks
				Drinking	Task(s)	
**RCT**						
Clerkin et al. (29)	AUD M(a) 44.3 (10.9)	EG1 Alc AtBM + soc. anx. Control (20) EG2 Alc control + soc. Anx. AtBM (24) EG3 Alc AtBM + soc. anx. AtBM^1^ (22) CG Alc control + soc. Anx. Control (20)	VPT-AtBM eight sessions	**FU** (1 week 1 month): ×	**VPT** ^2^: ×	^1^ Effects for social anxiety AtBM were also investigated, but not reported here. ^2^ AtB assessed traditionally as well as with measures separating different components of trial-level AtB
den Uyl et al. (30)	AUD M(a) 48.6 (0.9)	EG1 Control AtBM + active tDCS (20) EG2 AtBM + sham tDCS (20) EG3 AtBM +active tDCS (20) CG Control AtBM + sham tDCS (22)	VPT-AtBM four sessions	**FU** (1 year): ×	**VPT**: × **IAT**: ×	
Rinck et al. (27)	AUD M(a) 45.8 (9.5)	EG1 AtBM (230) EG2 combined ApBM and AtBM (255) CG sham + no training (682)	VPT-AtBM six sessions or VPT-AtBM three sessions and AAT-ApBM three sessions (combined)	**RR** (1 year): EG1: 44.8% < CG 55.6% EG2: 48.6% < CG 55.6% (trend, n.s.)	**VPT**: **Δ**AtB: EG1 < CG (trend, n.s.)^3^; EG2 < CG^3^ **AAT**: **Δ**ApB: EG1 > CG; EG2 > CG	Note that this study also included one ApBM group (as reported in **Table 1B**). ^3^ Training reduced the undesired increase of the bias that occurred in the control group Merged control groups (sham training and no training) for analyses
Schoenmakers et al. (31)	AUD M(a) 45.0 (9.9)	EG AtBM (21) CG control AtBM (22)	VPT-AtBM five sessions	**RR** (3 months): EG: 25% > CG: 21%^4^. EG on average 1.25 months longer until relapse compared to CG	**VPT**: EG: AtB↓^5^ CG:×	^4^ Difference not possible to statistically test due to low expected values in Chi^2^ test ^5^ Effects generalized to other stimuli, only visible for disengagement difficulties, not for rapid attention allocation
**ELS**						
Boendermaker et al. (32)	HD M(a) 21.2 (1.8)	EG1 regular (30) EG2 gaming (33) CG placebo (33)	VPT-AtBM four sessions	**FU** (2 weeks): AC and binge drinking:×	**VPT** (Go)^6^: EG1: AtB↓; EG2/CG: × **VPT** (Stay)^7^: × **Visual search task**: ×	^6^ Reflects rapid attention allocation ^7^ Reflects disengagement difficulties
Cox et al. (33)	Harmful drinkers M(a) 28.8 (14.4)	EG1 AtBM (35) EG2 LEAP (42) EG3 AtBM + LEAP (42) CG no intervention (29)	Stroop-AtBM four sessions	**Post-intervention**: AC^8^ ×, AC^9^ × **FU** (3 months): EG1: AC^8^↓, AC^9^↓ **FU** (6 months): EG1: AC^8^ ×, AC^9^↓	–	Note that effects were also investigated for a motivational intervention (LEAP; not reported here) ^8^ Mean weekly drinking ^9^ Atypical weekly drinking
Fadardi and Cox (34)	SD/HZD/HFD M(a) 32.6 (11.1)	EG1 SD (40) EG2 HZD (68) EG3 HFD (92)	Stroop-AtBM EG1 zero sessions EG2 two sessions EG3 four sessions	**FU** (3 months): EG3: AC↓, EG1/EG2: no FU assessed	**VPT**: EG1: AtB↑ EG2: AtB↓ **Alc Stroop**: EG2/EG3: AtB↓^10^ EG1. i.e. ×	No control group: Different drinking subsamples with a different number of sessions ^10^ Generalized to new stimuli
Field and Eastwood (35)	Heavy social drinkers M(a) 22.1 (3.9)	EG avoid^11^ (20) CG attend (20)	VPT-AtBM one session	**TT**: CG > EG	**VPT**: EG↓, CG↑	^11^ Control condition consisted of active training in the opposite direction
Field et al. (10)	HD M(a) 23.1 (8.7)	EG avoid (20) CG1 attend (20) CG2 unspecific exposure (20)	VPT-AtBM one session	**TT**: ×	**VPT**: EG: AtB↓^13^; CG1: AtB↑^12^; CG2:× **Flicker task**: × **Alc Stroop**: × **SRC**: ×	^12^ Effect generalized to novel stimuli ^13^ Effects did not generalize to different stimuli. In fact: AtB↑ for new stimuli
Langbridge et al. (36)	Binge drinkers Median age across all groups: 22	EG1 SOC + AtBM (10) EG2 No SOC + AtBM (10) EG3 SOC, no AtBM (10) CG1 no intervention (11) CG2 Non-binge drinkers, no intervention (10)	VPT-AtBM one session	**TT**: EG1 < CG1/CG2^14^	**VPT**: ×	^14^ AtBM alone unable to decrease alcohol consumption
Lee and Lee (37)	Problem drinkers M(a) 22.0 (2.6)	EG AtBM (21) CG psycho-education (22)	VPT-AtBM one session	–	**Eye-tracker**: EG: avoidance inclination↑ approach inclination↓^15^ CG: ×	^15^ Effects generalized to other stimuli
Luehring-Jones et al. (38)	Young adult drinkers M(a) 22.0 (2.2)	EG AtBM (30) CG sham (30)	VPT-AtBM one session	–	**VPT**: EG: AtB↓ **IAT** ^16^: EG: alc avoid↑ **Alc Stroop**: EG: AtB↓	^16^ Calculated with alcohol-incongruent reaction times (instead of overall bias score)
McGeary et al. (39)	HD M(a) 19.0 (1.1)	EG AtBM (19) CG control AtBM (22)	VPT-AtBM eight sessions	**Across the course of the 4-week training**: EG: AC↓; CG: ×	–	
Schoenmakers et al. (40)	HD M(a) 21.4 (2.0)	EG AtBM (53) CG no intervention (53)	VPT-AtBM one session	**Preference test** ^17^ ×	**VPT**: EG: AtB↓^18^ CG: × **Flicker task**: ×	^17^ Primed with a sip of beer. ^18^ Effects did not generalize to other stimuli
Wiers et al. (41)	Problem drinkers M(a) 47.4 (no SD)	EG AtBM (17) CG control ApBM^19^ (24)	Stroop-AtBM four sessions	**FU** (1–2 weeks, 1 month, 3 months): ×	–	Note that this study also included three ApBM groups (as reported in **Table 1B**). ^19^ No AtBM control condition

AC, alcohol consumption; alc, alcohol; AUD, individuals with Alcohol Use Disorder; AtB, Attentional Bias for alcohol stimuli; AtBM, Attentional Bias Modification; CG, control group; EG, experimental group; FU, alcohol consumption at follow-up; HD, heavy drinkers; HFD, harmful drinkers; HZD, hazardous drinkers; n, number of subjects; n.s., not statistically significant; M(a), mean age of sample; RR, relapse rate; SD, social drinkers or standard deviation (in column); SOC, Sense of control; soc. anx., social anxiety; SRC, stimulus-response compatibility task; TT, alcohol consumption during taste test; VPT, visual probe task; *×* = intervention showed no effect on outcome; *↑* = increase; *↓* = decrease; *→* = changed to; < = … less than…; > = …more than…. ΔAtB = Change in AtB from pre- to post-measurement. Mean age and standard deviation across all groups, as reported in paper or calculated as weighted mean/standard deviation (rounded to the first figure after the decimal). Reading example: In the study by Schoenmakers et al. (2007), the column “Drinking” shows that in a preference test, no group differences were found. The column “Task(s)” indicates that a VPT and flicker task were assessed. The VPT showed a reduction in attentional bias in the experimental group, whereas no effect were observed in the control group. The flicker task showed no effects.

**Table:** 

Table 1B: Approach Bias Modification (ApBM) studies							
Reference	Sample/mean age (SD)	Experimental (EG) and control (CG) groups (n)	Intervention/# sessions	Outcomes			Remarks
				Drinking	Task(s)	Neuronal activity	
**RCT**							
den Uyl et al. (42)	AUD M(a) 47.0 (8.8)	EG1 ApBM + tDCS (30) EG2 ApBM + sham tDCS (30) EG3 ApBM + sham tDCS + tDCS separate (31)	AAT-ApBM and tDCS four sessions	**RR** (3 months, 1 year)^1^	**AAT**: All groups ApB↓^2^	–	^1^ No control group without active ApBM, thus no conclusion possible on ApBM’s effects on relapse rates ^2^ No control group without active ApBM
Eberl et al. (26)	AUD M(a) 46.0 (9.0)	EG ApBM (248) CG no intervention (227)	AAT-ApBM 12 sessions	**RR** (1 year): EG 48.8% < CG 57.3%^3^	**AAT**: EG: ApB→AvB, CG: ×	–	^3^ Change in AAT mediates training effect on relapse rate
Loijen et al., 2018 (43)	AUD with alcohol-induced neuro-cognitive disorders M(a) 51.9 (15.6)	EG1 mild neurocognitive disorder (51) EG2 Korsakoff syndrome (54)	AAT-ApBM six sessions	–	**AAT**: ApB→AvB^4^	–	No control group without active ApBM ^4^ Note that the avoidance tendency evaluated within training performance from session one through six
Manning et al. (44)	AUD M(a) 40.0 (no SD)	EG ApBM (41) CG sham (42)	AAT-ApBM four sessions	**RR** (2 weeks): EG 31.4%. < CG 52.8% (trend, n.s.)^5,6^	–	–	^5^ Relapse rates calculated from continuous abstinence rates reported in paper ^6^ Sub-analyses of mean drinking days, mean standard drinks per drinking day, and time to relapse: ×
Rinck et al. (27)	AUD M(a) 45.7 (9.4)	EG1 ApBM (238) EG2 combined ApBM and AtBM (255) CG sham + no training (682)	AAT-ApBM six sessions (EG1) or VPT-AtBM three sessions and AAT-ApBM three sessions (EG2)	**RR** (1 year): EG1 47.9% < CG 55.6%; EG2 48.6% < CG (trend, n.s.)	**AAT**: **Δ**ApB: EG1 > CG (trend, n.s.); EG2 > CG **VPT**: ×; EG2 < CG^7^		^7^ Training reduced the undesired increase of the bias that occurred in the control group Note that this study also included one AtBM group (as reported in **Table 1A**). Merged control groups (sham training and no training) for analyses
Wiers et al. (28)	AUD M(a) 45.3 (8.0)	EG (implicit, explicit) (108) CG (sham, no intervention)^8^ (106)	AAT-ApBM four sessions	RR (1 year): EG 46% < CG 59% (trend, n.s.)^9^	**AAT**: ΔApB: EG > CG^10^ **IAT**: ΔApB: EG > CG	–	^8^ Groups were combined to one EG and one CG ^9^ Mediation by IAT or AAT scores could not be shown ^10^ Effects generalized to other stimuli
Gladwin et al. (45) [re-analysis of Wiers et al. (28)]	AUD M(a) 45.3 (8.0)	EG ApBM (108) CG sham (106)	AAT-ApBM four sessions	-^11^	**IAT**: **Δ**ApB: EG1 > CG^12^	–	^11^ EG: stronger AvB for alcohol-related stimuli mediated a decreased relapse probability ^12^ Note that stimulus-specific IAT scores on error rates were used
Wiers et al. (46)	AUD M(a) 44.0 (7.6)	EG ApBM (15) CG sham (17)	AAT-ApBM six sessions	–	**AAT**: ×	**fMRI** ^13^: EG: Amy↓^14^, Nac × CG: Amy ×, NAc: ×	^13^ Region of interest (ROI) analysis during cue reactivity task ^14^ Change correlated with change in subjective craving
Wiers et al. (47)[sample of Wiers et al. (46)]	AUD M(a) 43.9 (no SD)	EG (13) CG sham (13)	AAT-ApBM six sessions	–	**AAT**: ×	**fMRI** ^15^: EG: mPFC↓^16^, NAc × CG: mPFC ×, NAc: ×	^15^ Region of interest (ROI) analysis during AAT ^16^ Change correlated with decrease in ApB
**ELS**							
Claus et al. (48)	HD M(a) 24.5 (2.7)	EG1 ApBM + tDCS (23) EG2 sham ApBM + tDCS (20) EG3 ApBM + sham tDCS (16) CG sham ApBM + sham tDCS (20)	AAT-ApBM and tDCS four sessions	**FU** (1 week, 1 month): ×	**AAT**: ×	–	–
den Uyl et al. (49)	HZD M(a) 21.8 (3.2)	EG1 ApBM + tDCS (19) EG2 ApBM + sham tDCS (20) CG1 sham training + tDCS (19) CG2 sham training +sham tDCS (20)	AAT-ApBM and tDCS three sessions	**FU** (1 week, 1 month): ×	**AAT**: × **IAT**: ×	**EEG**: P300: ×	–
Di Lemma and Field (50)	HD M(a) 20.4 (2.1)	EG ApBM (30) CG sham (30)	AAT-ApBM one session	**TT**: EG < CG	**AAT**: × **IAT**: ×	–	Note that this study also included an IT experimental and control group (as reported in **Table 1C**)
Hahn et al. (51)	High risk young adults M(a) 20.0 (1.5)	EG ApBM (46) CG sham (45)	AAT-ApBM four sessions	**FU** (3 months): ×; **FU** (3 months): ×	**AAT**: EG: ApB↓ **AAT** (3 months): ×	–	ApBM used to simultaneously decrease maladaptive (i.e. alcohol use) and increase healthy protective behaviors (i.e. condom use). Only alcohol-related effects reported here.
Leeman et al. (52)	HD 21–25 years old	EG ApBM (35) CG sham (34)	AAT-ApBM four sessions	**Post-intervention**: AC ×	**AAT** : ×	–	
Lindgren et al. (53)	Study 1: social drinkers M(a) 20.5 (1.4). Study 2: At-risk drinkers M(a) 20.5 (2.1)	Study 1: EG ApBM (54) CG sham (46) Study 2: EG ApBM (47) CG sham (43)	AAT-ApBM two sessions	–	Study 1: **AAT**: × **IAT**: × Study 2: **AAT**: × **IAT**: ×	–	Also included general identity and personalized identity training (not reported here)
Sharbanee et al. (54)	Social drinkers M(a) 19.4 (2.1)	EG1 avoid (25) CG1 approach (25) CG2 sham (24)	AAT-ApBM one session	**TT**: × (trend, n.s.)^17^	**AAT**: ApB CG1 > CG2, EG1 < CG2; **Selective attention**: ×	–	^17^ Changes in AC mediated by ApB but not selective attention One control condition consisted of active training in the opposite direction
Wiers et al. (41)	Problem Drinkers M(a) 48.3 (no SD)	EG1 ApBM-Explicit (27) EG2 ApBM-Implicit100 (35) EG3 ApBM-Implicit90 (33) CG control ApBM (24)	AAT-ApBM four sessions	**FU** (1–2 weeks, 1 month, 3 months): ×	–	–	Note that this study also included an AtBM group (as reported in **Table 1A**)
Wiers et al. (55)	HZD aged 18–28	EG avoid (21) CG approach (21)	AAT-ApBM one session	**TT**: Whole group ×. Successful trainers^18^: EG: AC↓CG: AC↑	**AAT**: EG: ApB→AvB^19^ CG: × **IAT**: EG AvA↑ CG: ×	–	^18^ Note that only the subgroup “successful trainers” showed significant effects in the expected direction. ^19^ Effects generalized to other stimuli

AAT, Approach-Avoidance Task; alc, alcohol; Amy, amygdala; AC, alcohol consumption; ApA, alcohol approach association; ApB, alcohol approach bias; ApBM, Approach Bias Modification; AtBM, Attentional Bias Modification; ATT, action tendency task; AUD, individuals with Alcohol Use Disorder; AvA, alcohol avoidance association; ApB, alcohol approach bias; AvB, alcohol avoidance bias; CG, control group; EG, experimental group; fMRI, functional magnet resonance imaging; FU, alcohol consumption at follow-up; HZD, hazardous drinkers; IAT, implicit association task; IT, Inhibition Training; M(a), mean age of sample; mPFC, medial prefrontal cortex; n = number of subjects; NAc, nucleus accumbens; n.s., not statistically significant; RR, relapse rate; SD, social drinkers or standard deviation (in column); tDCS, transcranial direct-current stimulation; TT, alcohol consumption during taste test; × = intervention showed no effect on outcome; *↑* = increase; *↓* = decrease; *→* = changed to; < = … less than…; > = …more than…. ΔApB = Change in ApB from pre- to post-measurement. Mean age and standard deviation across all groups, as reported in paper or calculated as weighted mean/standard deviation (rounded to the first figure after the decimal).

Reading example: In the study by Wiers, Rink et al. (2010, lowest row), the column “Tasks” shows that an AAT and an IAT were assessed. Results in the AAT showed that from pre- to post- intervention, EG shifted from Approach (ApB) to Avoidance bias (AvB), while CG showed no effects. In the IAT, EG showed an increase in alcohol avoidance association (AvA), while CG showed no effects.

**Table:** 

Table 1C: Inhibition Training (IT) studies							
Reference	Sample/mean age (SD)	Experimental (EG) and control (CG) groups (n)	Intervention /# session	Outcomes			Remarks
				Drinking	Task(s)	Neuronal activity	
**ELS**							
Bowley et al. (56)	(no definition) M(a) 20.8 (2.0)	EG1 BeerNoGo (20) CG1 BeerGo^1^ (20) CG2 BAI (19)	GNG training/BAI one session	**TT**: EG1/CG2 < CG1 **FU** (1 week): ×	**IAT**: ×^2^	**EEG**: EG1/CG2: × CG1: frontal activity in left hemisphere↑ (trend, n.s.)	^1^ Control condition consisted of active training in the opposite direction ^2^ However: positive relationship between implicit beer-related cognitions at post-training and AC in TT (trend, n.s.)
Di Lemma and Field (50)	HD M(a) 20.3 (2.0)	EG Alc NoGo (30) CG sham (30)	GNG training one session	**TT**: EG < CG	**GNG**: × **IAT**: ×	–	Note that this study also included an ApBM experimental and control group (as reported in **Table 1B**)
Houben et al. (57)	HD M(a) 22.4 (4.9)	EG BeerNoGo (25) CG BeerGo^3^ (27)	GNG training one session	**TT**: EG: AC↓ (trend, n.s.); **FU** (1 week): EG: AC↓ CG: AC↑	**IAT**: EG: positive implicit attitude↓ CG: ×	–	^3^ Control condition consisted of active training in the opposite direction
Houben et al. (58)	HD M(a) 20.9 (1.8)	EG BeerNoGo (27) CG BeerGo^4^ (30)	GNG training one session	**FU** (1 week): EG: AC↓ CG: ×^5^	**SST**: × **IAT**: EG: positive implicit attitude↓ CG: × **SRC**: ×	–	^4^ Control condition consisted of active training in the opposite direction ^5^ Effect of the GNG training on alcohol intake mediated by training-induced changes in IAT scores
Jones and Field (59)	Heavy social drinkers M(a) 20.8 (2.7)	EG alcohol restraint (30) CG1 no training (30) CG2 neutral restraint (30)	SST training one session	**TT**: EG < CG1/CG2 **FU** (1 week): ×	**SST**: inhibition errors to alcohol cues: EG↓, CG2↑^6^	–	^6^ Measured form first to last block within the one training session
Jones et al. (60)	HD M(a) 41.3 (11.7)	EG1 Alcohol NoGo (57) EG2 Alcohol stop signal (60) EG3 General inhibition (58) CG active control (54)	GNG and SST training 8–14 sessions	**During intervention**: × **FU** (2-, 4-, and 6-week): ×	**Alcohol SST**: × **General SST**: × **IAT**: ×	–	
Kilwein et al. (61)	HD M(a) 22.6 (2.1)	EG alcohol NoGo (23) CG water NoGo (21)	GNG training one session	**TT**: ×^7^ **FU** (2 weeks) EG: AC↓ **CG**: AC↑	**SST**: × **IAT**: × **SRC**: ×	–	^7^ But EG took significantly longer to take the first sip
Liu et al. (62)	Regular Drinkers aged 18–30	Total N = 88 EG Beer NoGo CG1 Beer Go CG2 Oddball CG3 BAI	GNG training one session	**TT**: EG < CG1–3 **FU** (1 week): EG: AC↑. CG1–3: AC↓ **FU** (4 weeks): ×	–	–	
Smith et al. (63)	Regular Drinkers M(a) 21.7 (0.7)	EG1 BeerNoGo (24) EG2 restrained stop (22) EG3 combined (22) EG4 BAI (24) CG neutral control task (22)	GNG, GNG variation, or BAI one session	**TT**: EG3: Relative amount of beer↑ **FU** (1 week): ×^8^	**IAT**: × **Flanker Task**: ×	–	^8^ Only BAI but not IT successful at reducing the number of standard drinks of beer and the number of drinking days per week compared to CG
Strickland et al. (64)	AUD M(a) 34.4 (9.7)	EG1 Ihibitory control (138) CG1 Working memory^9^ (132) CG2 (132)	GNG training14 sessions	**Post-intervention**: AC × **FU** (2 weeks): AC^10^ × AC^11^: EG↓ CG: ×	**GNG**: inhibition errors to alcohol cues: EG↓^12^	-	^9^ Effects for working memory training were also investigated, but not reported here Recruitment *via* online crowdsourcing platform: not treatment-seeking participants ^10^ Heavy drinking days ^11^ Drinking days ^12^ Measured form first to last block during training

AC, alcohol consumption; alc, alcohol; AUD, individuals with Alcohol Use Disorder; BAI, Brief Alcohol Intervention; CG, control group; EEG, electroencephalography; EG, experimental group; FU, alcohol consumption at follow-up; GNG, Go NoGo Task; HD, heavy drinkers; IAT, implicit association task; M(a), mean age of sample; n = number of subjects; n.s., not statistically significant; SD, standard deviation; SRC, stimulus response compatibility task; SST, stop signal task; TT, alcohol consumption during taste test; *×* = intervention showed no effect on outcome; *↑* = increase; *↓* = decrease; < = … less than…; > = …more than … Mean age across all groups, as reported in paper or calculated as weighted mean/standard deviation. Mean age and standard deviation across all groups, as reported in paper or calculated as weighted mean/standard deviation (rounded to the first figure after the decimal). Reading example: In the study by Strickland et al. (2019), the column “Drinking” shows no effects on alcohol consumption at post-intervention. At 2-week follow-up, no effects on heavy drinking days were observed, whereas drinking days decreased in the experimental but not in the control group. As for tasks, the experimental group showed a decrease in alcohol-related inhibition errors from the first to last training block.

### Quality Assessment

The quality of the included studies was assessed based on the Cochrane Collaboration’s Risk of Bias guidelines (65) by the first (HB) and last (FM) authors. Whenever unclear, the second author (MS) was included in the discussion until a conclusion was reached. All information is found in the **Supplementary Table 1**. Apart from Cochrane criteria, specific methodological factors were also extracted, so that the table contains the following information:

1. Sample composition (e.g. students only, general population, or clinical sample), mean age (+ standard deviation), sex ratio (% female participants)
2. Number of sessions and setting (e.g. lab or web/home-based intervention)
3. Whether motivation (regarded as moderator for CBM effectiveness) was assessed, and if so, if it was included in the analyses
4. Whether a power analysis was conducted prior to the study onset, and whether the calculated N was achieved
5. Adequate generation of allocation to conditions (randomization bias)
6. Blinding procedure (prevention of knowledge of the allocated intervention to participants and/or study member conducting the study: single- or double-blind design)
7. Attrition bias: Initial N and N for (sub-)analyses

## Results

**Figure 2:**
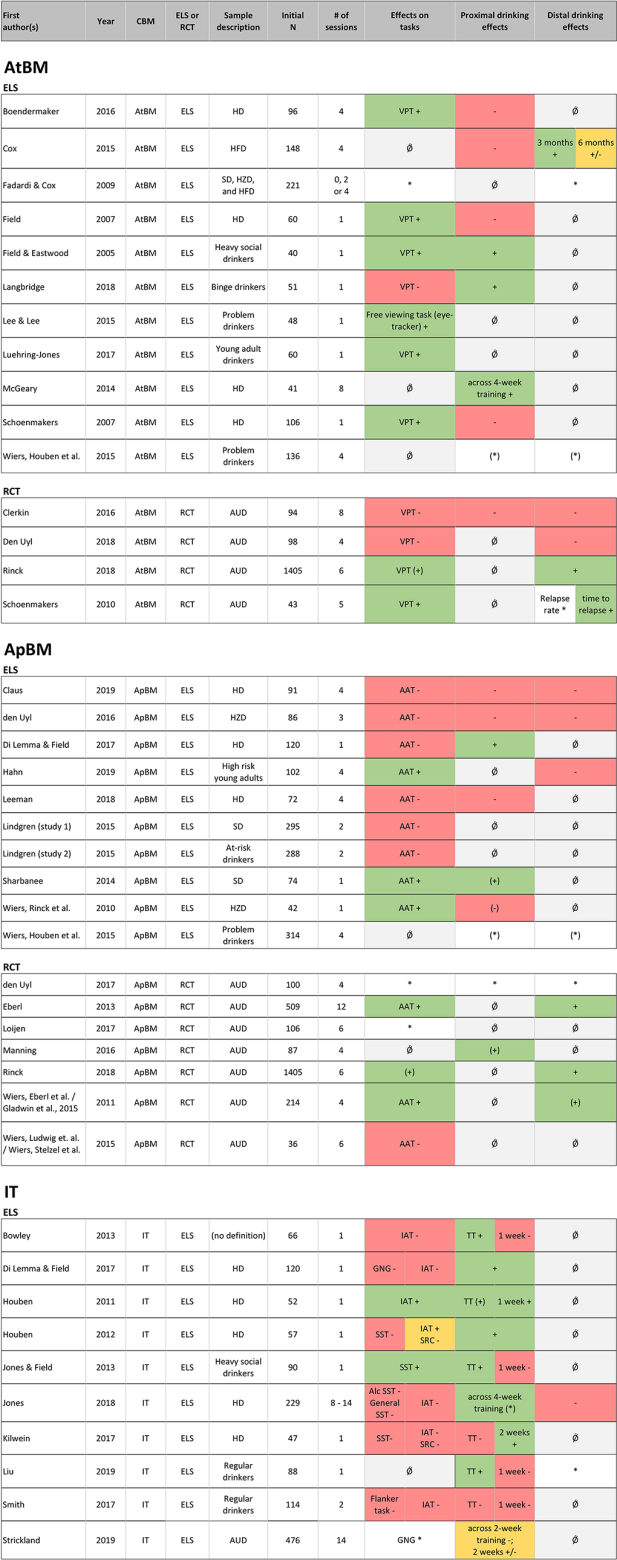
Alignment of studies according to CBM type (AtBM, ApBM, or IT) and study type [non-clinical experimental laboratory study (ELS) or clinical randomized-controlled trial (RCT)] and overview of effects on bias change/experimental tasks as well as proximal and distal drinking outcomes. *Notes*. This figure is organized according to CBM type [i.e. Attentional bias modification (AtBM), approach bias modification (ApBM) and inhibition training (IT)] and within the CBMs differentiated between experimental laboratory study (ELS) including non-clinical samples and randomized-controlled trials (RCT) including clinical samples. Effects are reported as follows, with color coding in parentheses: + significant effect (green); (+) trend (green); +/− mixed results (orange); − no effect of the intervention (red); (−) no effects in ITT analysis, but subgroup with bias change showed drinking reduction (red); * outcome assessed, but not statistically tested or no control group (white); (*) no clear effect of intervention, but pre-post reduction in all groups (including control group) (white); Ǿ not measured (gray). Effects are summarized using a conservative approach in the spirit of intention-to-treat analyses (whole group analyses) and only positive effects observed against a control group were seen as clear evidence. Proximal effects: up to 1 month; Distal effects: 1 month and more. AAT, Approach Avoidance Task; ApBM, Approach Bias Modification; AtBM, Attentional Bias Modification; AUD, Patients With Alcohol Use Disorders; CBM, Cognitive Bias Modification; ELS, Experimental Laboratory Study; GNG, Go/Nogo Task; HD, Heavy Drinkers; HFD, Harmful Drinkers; HZD, Hazardous Drinkers; IAT, Implicit Association Task; IT, Inhibition Training; RCT, Randomized-Controlled Trial; SD, Social Drinkers; SRC, Stimulus Response Compatibility Task; SST, Stop-Signal Task; TT, Taste Test; VPT, Visual Probe Task. Reading example: In those RCTs on ApBM, which measured both, bias change and drinking effects (26–28), one can see that the effects on bias change (column “bias change”) consistently point in the same (positive) direction as the effects on proximal and distal drinking outcomes. In the ELS studies on ApBM, not only is this pattern less clear, but one can also see that two-thirds of the studies failed to report a bias change in the first place.

**Figure 3:**
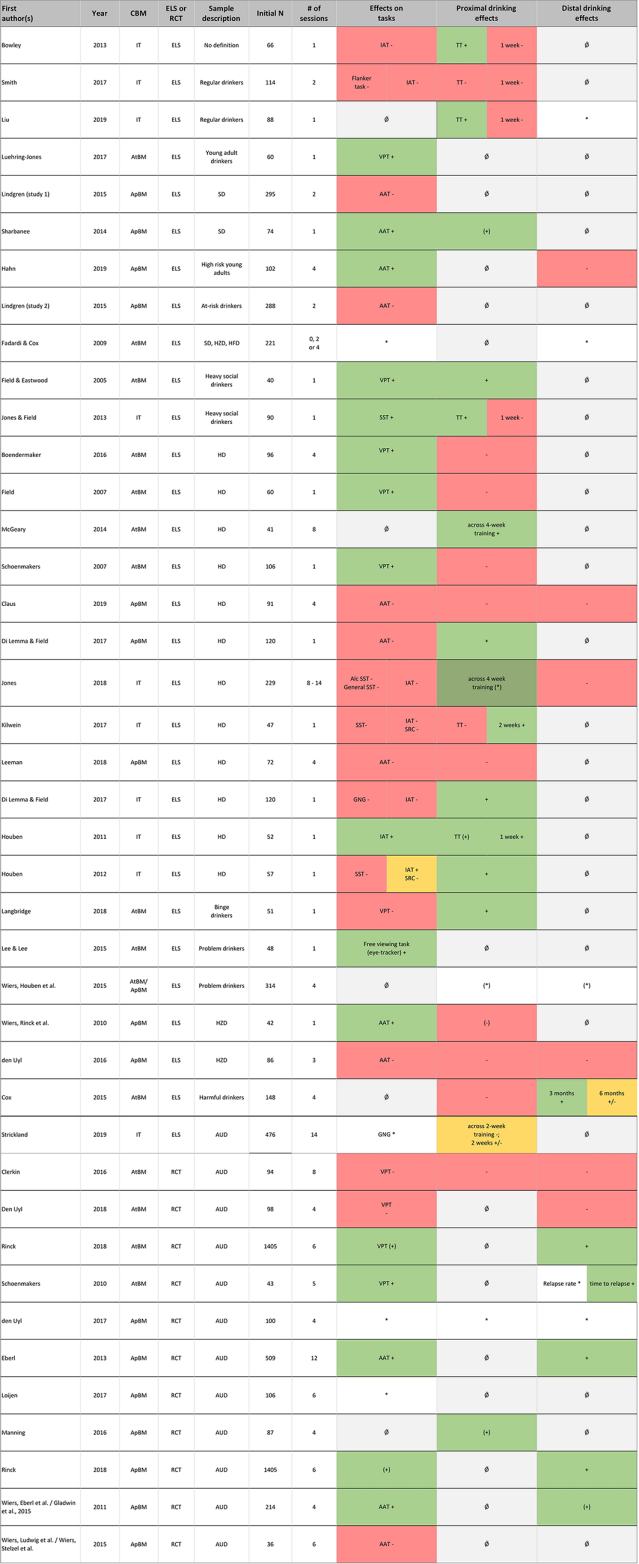
Alignment of all studies according to description of the samples’ severity of drinking behavior and overview of effects on experimental tasks and proximal and distal drinking outcomes. *Notes*. Effects are reported as follows, with color coding in parentheses: + significant effect (green); (+) trend (green); +/− mixed results (orange); − no effect of the intervention (red); (−) no effects in ITT analysis, but subgroup with bias change showed drinking reduction (red); * outcome assessed, but not statistically tested or no control group (white); (*) no clear effect of intervention, but pre-post reduction in all groups (including control group) (white); Ǿ not measured (gray). Effects were summarized using a conservative approach in the spirit of intention-to-treat analyses (whole group analyses) and only positive effects observed against a control group were seen as clear evidence. Proximal effects: up to 1 month; Distal effects: 1 month and more. AAT, approach avoidance task; ApBM, Approach bias modification; AtBM, Attentional bias modification; AUD, patients with alcohol use disorders; CBM, cognitive bias modification; ELS, experimental laboratory study; GNG, Go/NoGo task; HD, heavy drinkers; HFD, harmful drinkers; HZD, hazardous drinkers; IAT, implicit association task; IT, inhibition training; RCT, randomized-controlled trial; SD, social drinkers; SRC, Stimulus response compatibility task; SST, stop-signal task; TT, taste test; VPT, visual probe task. Reading example: In the RCTs summarized in the lower part of the figure, one can see that the effects on bias change (column “bias change”) mostly point in the same direction as the effects on proximal and distal drinking outcomes. In the ELS studies, this pattern is inconsistent.

Results are summarized first for each CBM separately, before an overall summary is given, which also addresses aspects of potentially moderating variables, such as drinking problem severity and motivation to change alcohol consumption. Both a detailed summarizing table (**Table 1**), and synoptic figures (**Figures 2** and **3**) accompany this summary; in **Table 1**, important details regarding the characteristics and results of each study are listed. **Figures 2** and **3** offer a synoptic depiction indicating which study has yielded positive or negative results with respect to bias and drinking behavior change (note that **Figure 2** is aligned according to CBM type while **Figure 3** is aligned according to drinking problem severity).

### Attentional Bias Modification (AtBM)

AtBM aims to alter AtB by training to direct attention away from alcohol-specific cues (40). The intervention is typically based on the visual probe task (VPT), during which alcohol-specific and control pictures appear simultaneously on a computer screen. When they disappear, a visual probe appears in the location of one of the previously shown pictures, and participants are required to react accordingly (35). During the VPT-AtBM intervention, the visual probe consistently replaces the control pictures, thus training participants to direct their attention to these pictures and away from the alcohol-related pictures. Such an AtBM intervention is then usually compared to a control condition, during which probes are divided equally between alcohol-specific and neutral stimuli. Certain other studies, however, feature different control conditions (e.g. 31, 35).

Some studies (33, 34, 41) based their intervention on a Stroop-like rationale (Stroop-AtBM) aiming to train participants to ignore the task-irrelevant aspect of stimuli (e.g. alcohol-relatedness), and to respond faster to another aspect (e.g. the color). **Table 1A** summarizes twelve studies investigating alcohol-specific computerized AtBM.

### AtBM: Effects on Experimental Tasks

Research investigating the effects of VPT-AtBM has reported changes in VPT-AtB in the expected direction in young adult drinkers (38), heavy social drinkers (35), heavy drinkers (HD) (10, 32, 40), and AUD (31). Another study in AUD showed a trend (27), while two studies in AUD (29, 30), and one in binge drinkers (36) found no effects. It is also noteworthy that in Rinck et al. (27), neither baseline bias nor bias change did mediate drinking changes. In the seven studies investigating the generalization to other tasks, four reported no generalization (e.g. the Stroop (10), the flicker (10, 40), the stimulus response task (10), the implicit association task (30) or the visual search task [32)]. Meanwhile, one study found VPT-AtBM to inflict expected changes in both an implicit association task and an alcohol-specific Stroop task (38). Further, two studies reported an effect of AtBM on alcohol approach inclination as measured by an approach-avoidance test (27) or by eye-movements in a free viewing task (37). When employing Stroop-AtBM, which is inspired by, but not identical to a Stroop paradigm, effects on an alcohol-related Stroop task were reported for hazardous and harmful drinkers (34). This might be interpreted as evidence for generalization to a new—albeit similar—task. The sole study that investigated the persistence of AtBM effects over time reported that the effects of a Stroop-AtBM on an alcohol-related Stroop-task could still be found at 3-month follow-up (34).

### AtBM: Effects on Drinking Behavior

Regarding the effect of VPT-AtBM on alcohol consumption in experimental lab studies, two studies reported less drinking during (39) or immediately after the intervention period (35), while three studies failed to observed such effects in non-clinical samples immediately (10, 36) or 2 weeks after the intervention (32). In a so-called preference test, which does not measure drinking behavior since participants simply choose between alcoholic and non-alcoholic beverages without actually drinking them, no differences were found either (40).

Two studies reported a decrease in alcohol consumption in non-clinical samples 1 week or 3 months after Stroop-AtBM, however these studies either lacked a control group (34) or the effect was observed in all groups (41). Taken together, experimental laboratory studies do not suggest that AtBM reliably changes drinking behavior. It has been argued that this might be attributable to a lack of motivation to change drinking behavior. However, the only experimental lab study which included non-clinical harmful drinkers motivated to cut down their drinking only partially aligns with this assumption: In this study investigating the effects of Stroop-AtBM on drinking behavior, effects were either marginal or not significant at post-intervention. Nevertheless, at later time points, there were significant effects on different measures of alcohol consumption at 3-month follow-up, with effects partially persisting even until 6 months after the intervention (33).

Generally, motivation to change is assumed to be higher in populations with more problematic drinking patterns. When considering clinical trials on AtBM in AUD, however, two of four studies reported that AtBM increases the time to first drink at 3-month follow-up (31) or reduces relapse rates at 1-year follow up (27), while two other studies failed to report such effects at 4-week (29) or 1-year follow-up (30). Interestingly, those studies, which reported evidence for a bias change (27, 31) were also those reporting drinking effects, while the studies, in which the bias could not be changed did not find any effects on drinking behavior (29, 30).

### AtBM: Effects on Neuronal Activity

To the best of our knowledge, there are no studies investigating neurophysiological effects of AtBM.

### Summary and Conclusion: Attentional Bias Modification (AtBM)

Considering effects on experimental tasks, two-thirds of the studies showed that AtBM influences AtB when measured with a similar task. Support for generalization to other cognitive tasks is limited, with only one-third of the studies reporting such effects. It is possible that these tasks measure different and only slightly intercorrelated incentive-motivational aspects of alcohol-related cues that remain unaffected by the intervention.

Beneficial effects of AtBM on drinking behavior were only observed in the minority of experimental lab studies. In clinical RCTs, where the motivational prerequisites for the translation of bias to drinking behavior change might be given more often, the two studies observing a bias change also observed changes in drinking behavior, while the two studies that failed to change the bias reported no drinking changes. Note however, that the only study analyzing specifically whether bias change mediates changes in drinking behavior did not show such a mediation (27).

It has been suggested that drinking behavior change should only be expected if a bias change was achieved and if participants are motivated to change their drinking behavior (24). Considering that, one might argue that the reported “synchrony” in bias change and drinking change is in line with this expectation, but the failure to observe a mediation effect in 27 speaks against such a conclusion. Challenging this view more generally, Cristea et al. (66) have argued that it is questionable to select only the studies with successful bias change when it comes to the summary of effects on drinking behavior.

Regardless of this controversy, it remains to state that in clinical trials, the intended effects on bias change or drinking behavior, respectively, could not be reliably replicated. Factors influencing this variability must be identified, experimentally examined, and optimized if AtBM is to truly help clinical populations with AUD.

### Approach Bias Modification (ApBM)

Alcohol approach/avoidance inclinations can be assessed with the Approach Avoidance Task (AAT), which has also been the basis for the development of an intervention targeting action tendencies (55). In this ApBM intervention, pictures of alcoholic and non-alcoholic beverages are presented. Depending on the format (landscape or portrait), participants must react either by avoiding the cue, i.e. pushing a joystick away from themselves, or approaching the cue, i.e. pulling a joystick toward them (55). In the AAT, which is used to assess action tendencies, alcohol-related pictures appear with equal probability in either landscape or portrait format. The differences in reaction time between the push and the pull trials reflect approach/avoidance biases. In the training version to modify AAT (AAT-ApBM), however, the picture type is linked to the picture format: Aiming to decrease alcohol approach tendencies, alcohol-related pictures appear in the format that required participants to avoid the stimulus. In non-clinical trials, opposite contingencies, i.e. pairing alcohol-related pictures with the approach reaction, potentially increasing ApB, are often used in control conditions. Because such a condition is less favorable in AUD patients, clinical studies have used control conditions using either equal probabilities for the alcohol-push and -pull-pairings (e.g. 28) or neutral images (e.g. 44). **Table 1B** summarizes 15 papers investigating alcohol-specific computerized ApBM.

### ApBM: Effects on Experimental Tasks

ApBM was shown to modify alcohol avoidance and approach bias (AvB/ApB) in the expected direction in social drinkers (54), hazardous drinkers (HZD) (55), risky drinkers (51), and AUD (26–28). However, replication of these effects failed in six studies in social (53), heavy (48, 50, 52, 53), hazardous drinkers (49), and AUD (42, 46). Loijen et al. (43) reported an ApB reduction in AUD but did not test this effect against a control group, thereby limiting the statistical explanatory power.

Regarding generalization of effects to new tasks, two studies showed a generalization to the IAT (28, 55). Yet, three studies conversely found no effect on the IAT (49, 50, 53), neither did these same studies find an effect on ApB in the first place. Sharbanee et al. (54) reported generalization to another measure of ApB, but not to a measure of selective attention.

With respect to working mechanisms, there is some evidence that the effect of ApBM on ApB is moderated by strong baseline ApB (26), and that the training effect on drinking outcomes is mediated by change in ApB (26, 54, 55). However, this mediation effect could neither be replicated for baseline ApB nor for change in ApB in a clinical trial by Rinck et al. (27). In another clinical sample, neither change in ApB scores nor change in general IAT scores mediated the effect of ApBM on relapse rates (28), while alone stimulus-specific IAT scores did (45).

### ApBM: Effects on Drinking Behavior

Experimental lab studies conducted in non-clinical samples supposedly not motivated to change drinking behavior reported no effects on drinking during a post-intervention taste test in social (54), heavy (52) or hazardous drinkers (55). However, additional analyses in two of these studies (54, 55) indicated that successfully trained participants, whose ApB/AvB scores changed in the intended direction, showed the expected alterations in drinking behavior. Additionally, one study did find HD to drink significantly less than controls during a taste test (50). Four studies with hazardous and heavy drinkers observed no effects of ApBM on alcohol consumption at 1-week, 1-month, or 3-month post-intervention (41, 48, 49, 51).

Contrary to these findings in experimental lab studies, the majority of clinical RCT trials did report effects of ApBM on drinking behavior. One study in AUD showed a 21.4% lower relapse rate in the ApBM compared to the control group at 2-week follow-up (marginally significant), but did not report differences regarding time to relapse, mean drinking days, and mean standard drinks per drinking day (44). Long-term effects of ApBM on drinking behavior were analyzed in three clinical studies with AUD samples, with clinically relevant reductions in relapse rates at 1-year follow-up of 13% (marginally significant) (28), 8.5% and 7.7% (26, 27, both significant). The pattern of results in ApBM thus aligns with the assumption that the translation of bias change to effects on drinking behavior works better in clinical samples, assumedly because patients are more motivated to reduce drinking. However, none of the clinical trials on ApBM actually measured their participants’ motivation to change.

### ApBM: Effects on Neuronal Activity

fMRI studies showed ApBM to decrease amygdala activation during a cue-reactivity task, an effect also related to decreased subjective craving (46). Comparing brain activation during the AAT from pre- to post-intervention, ApBM reduced activation in the medial prefrontal cortex (mPFC), specifically in the dorsal anterior cingulate cortex (dACC) and middle frontal gyrus. The decrease in dACC was also positively correlated to ApB changes (47). By contrast, the only electrophysiological study examining the P3 peak amplitude during an oddball and cue reactivity task yielded no significant effects (49).

### Summary and Conclusion: Approach Bias Modification (ApBM)

Analyses on experimental data is mixed: about half of the studies report an effect of ApBM on ApB while the other half does not. Thus, compared to earlier reviews (e.g. 24), this systematic review of all currently available data suggests a more cautious evaluation of the potential of ApBM to impact ApB, underlining the necessity to clarify under which circumstances ApBM can change the targeted bias. To date, it seems that bias change can be more reliably achieved in clinical populations (three out of four studies) than in non-clinical experimental lab studies (three out of eight studies). This might be due to clinical populations showing a stronger ApB at baseline, which has been shown to moderate ApBM effects (26, but see 27), while ApB baselines are rarely assessed. Remarkably, if other tasks were included in the study, those studies observing positive effects on ApB could also report generalization of effects. This pattern is consistent with mediation analyses and earlier reviews suggesting that ApBM achieves its beneficial effects *via* ApB changes (18, 26, 45, 54, 67), even if other studies did not find such mediation (27 and 28).

In clinical populations, ApBM reduced alcohol consumption and relapse rates in AUD (26–28, 44), the limitation being that some of these effects are marginally significant. Nevertheless, since they comprise remarkable effects on relapse rates, they are considered to be of clinical relevance. The majority of studies in non-clinical samples showed no effects on drinking behavior even after multiple sessions of ApBM (41, 42, 54, 55). This discrepancy between clinical and non-clinical samples is discussed in terms of motivational differences (17). It might, however, also be due to the difficulty in achieving a bias change in non-clinical populations in the first place. The proposed working mechanism suggests that a change in drinking behavior should only be expected if the according bias alteration could be achieved first. In line with this assumption, all the clinical trials investigating bias changes and reporting beneficial effects on drinking behavior could also show a bias change (see **Figure 2**). In the seven pre-clinical experimental laboratory studies investigating both bias change and drinking outcomes, four reported consistent (non-)effects (i.e. bias change and drinking behavior change, or no bias change and no drinking behavior change), with one study reporting a bias as well as a drinking change (54), and three showing neither a bias change nor a drinking change (48, 49, 52). One study (55) reported a bias but no drinking change in the whole group analysis, but reported a significant drinking reduction for those whose bias had been changed successfully by the intervention. In the two remaining studies, behavioral and cognitive effects diverge, with one reporting no bias change, albeit a reduction in drinking behavior (50) and one showing a bias but no drinking behavior change (51), the latter presumably explained by a lack of motivation to change.

Overall, we conclude that a) the evidence speaks for the proposed working mechanism of ApBM to work *via* changes in ApB and that b) this bias change as well as changes in drinking behavior could quite consistently be shown in clinical populations. However, c) effects regarding bias change as well as drinking behavior change are inconsistent in non-clinical experimental studies. Earlier reviews (e.g. 24) explained the lack of effects in drinking behavior in non-clinical populations with the lack of motivation to change. But this argument cannot be extended to explain the variation of effects regarding bias change, as this is supposed to be independent of motivation or even awareness. However, it is possible that variations in baseline ApB, which might differ between clinical and non-clinical samples, could explain this divergence.

Neurophysiological effects were reported using fMRI (46 47) but not EEG (49), and indicate that ApBM induces changes in regions related to saliency (Amygdala) and motivational values of stimuli (mPFC).

### Inhibition Training (IT)

Two tasks measuring different aspects of inhibitory control (68), the Go-NoGo-task (GNG, measuring action restraint) and the Stop signal task (SST, measuring action cancellation), served as a basis for the development of IT. To date, the majority of IT studies have based their training intervention on a modified GNG, in which alcohol-related stimuli are paired consistently with a stopping response. This alcohol-NoGo-intervention has either been contrasted with a control condition, in which alcohol-related stimuli are consistently paired with a Go response, or with control conditions utilizing only neutral stimuli in Go and NoGo trials. Alcohol-specific IT based on a modified SST was also developed, but to date tested in only one study (59): 59 used an SST-based alcohol-specific IT in which an auditory cancellation signal was consistently paired with alcohol-related stimuli. In the control conditions, cancellation was either paired with neutral cues or auditory cues were to be ignored.

The discussion about the potential working mechanisms and the conceptual framing of IT followed mainly two lines: One assumption is that IT improves inhibitory control over a patient’s reaction to alcohol-specific cues (69). Following this line, effects of IT would be expected in inhibitory control tasks. Another explanation, the stimulus devaluation hypothesis (70) suggests that IT is a means to decrease motivational properties of a stimulus. This hypothesis assumes that IT affects implicit semantic associations to alcohol-specific cues and has led to the definition of IT as a form of (implicit) memory bias alteration (24). In the section *IT: Effects on Experimental Tasks*, we will review whether existing data speaks in favor of one or the other hypothesis and whether the conceptual framing of IT as a form of memory bias modification is still justified. **Table 1C** summarizes ten studies investigating alcohol-specific computerized IT.

### IT: Effects on Experimental Tasks

Considering the mechanism of action of IT, two main hypotheses have guided the selection of experimental tasks employed in these studies. The first and more straightforward hypothesis is that IT increases inhibitory control (69) and should thus affect participant’s performance on tasks such as the GNG or the SST. The second hypothesis, the stimulus devaluation hypothesis (70), states that consistently pairing a stimulus with a stopping response decreases the stimulus’ valence and motivational properties, an effect observable in tasks such as the implicit association task (IAT).

The effects of alcohol-specific IT on inhibitory control have been investigated in six studies: Regarding SST-based IT, one study reported a selective decrease in inhibition errors in alcohol-related stop trials when comparing the first to the last training block which might indicate an improvement of inhibitory control (59), while another study reported no effects on the SST (60). Regarding GNG-based IT, four studies reported no effects on inhibitory control as indicated by an SST (50, 58, 60, 61) or by reaction times during Go-trials on a GNG (50). Strickland et al. (64) reported a decrease in errors of commission (EOCs) during training, but could not compare this finding to a control group. Notably, however, there is no data on whether GNG-based IT alters the number of EOCs during a GNG performed after training, even though these are a typical parameter of inhibitory control (6).

The effects of alcohol IT on implicit associations have been examined in seven studies. Two studies reported that GNG-based IT decreased positive implicit associations to alcohol (57, 58), supporting the stimulus devaluation hypothesis. Yet, five subsequent studies were unable to replicate this effect for GNG-based IT (50, 56, 60, 61, 63) or SST-based IT (60). As the IAT effect originally motivated the conceptual framing of IT as a form of memory bias modification, this conceptualization seems questionable in light of this current summary.

No generalization was observed on automatic action tendencies (58) nor on a stimulus response compatibility task (61).

### IT: Effects on Drinking Behavior

To date, effects on alcohol-specific IT on drinking behavior have so far only been investigated in non-clinical samples, and as such, have primarily included participants who lack the motivation to change their drinking behavior. Motivation to change is discussed to be a necessary prerequisite for any CBM to affect drinking behavior (24). Even if the sample in the Strickland et al. (64) study was required to fulfill the DSM-5 criteria for AUD, the participants were not seeking treatment. Moreover, less than 20% of the sample reported a desire to reduce drinking, which is why this study is classified as an experimental lab study rather than a clinical randomized controlled trial in this review. With respect to alcohol consumption measured in a taste test immediately after the intervention, four studies reported a decrease due to alcohol-specific IT (50, 56, 59, 62), one study reported a non-significant trend (57), and another observed no effect other than an unexpected increase in one of the experimental conditions (63). The sole effect in Kilwein et al. (61) was that it took the experimental group significantly longer to take the first sip of an alcoholic beverage. Across the 2 weeks of IT training, no reduction in alcohol consumption could be found in one study (64). When assessing alcohol consumption for 1 or 2 weeks post-intervention, three studies reported a decrease due to alcohol-specific IT (57, 58, 61). Interestingly, in two of these studies (57, 61) participants in the control condition also drank significantly more. One study (64) found a reduction of overall drinking days, but no effect on heavy drinking days. Three studies found no significant effects of IT on drinking behavior at 1-week follow-up (56, 59, 63), and in another study, the experimental group (Beer NoGo) targeted to reduce drinking even drank significantly more than the control group (Beer Go; 62). One study investigated the effects of IT in HD, who had reported to be motivated to reduce alcohol consumption, but found no significant effects on drinking across the 4-week study participation, nor at 2-, 4-, and 6-week follow-up (60). Lastly, Liu et al. (62) found a decrease of alcohol consumption at 4-week follow-up in all groups, including the control condition.

### IT: Effects on Neuronal Activity

To date, the only study investigating neurophysiological effects of IT in HD (56) concentrated on frontal EEG asymmetry in the alpha frequency band as an indicator of approach motivation. Results showed an insignificant change in frontal EEG asymmetry, corresponding to decreased approach motivation.

### Summary and Conclusion: Inhibition Training (IT)

Considering the effects on experimental tasks, the data currently contradicts earlier summaries (24, 67), and fails to support the notion that IT works *via* stimulus devaluation, at least not when assessed with the IAT (50, 56, 60, 61, 63). Thus, while earlier reviews conceptualized IT as a form of memory bias alteration (24), our current review shows that this conceptualization is contradicted by the available data and seems no longer justifiable. With respect to inhibitory performance, only one study (59) reported an immediate effect of IT on inhibitory performance, while five studies failed to report such effects when assessing inhibition with a SST (57, 58, 60), a flanker task (63), or with Go reaction times during a GNG (50). These results leave the question of how IT eventually works still unanswered. In the case of an inhibitory working mechanism, there are, however, two points to be considered: Firstly, there is a noticeable lack of data on a proximal experimental outcome of a GNG-IT, the analysis of errors of commission during a GNG. Secondly, most IT studies used Go-NoGo ratios of 50/50, making inhibition less strenuous, thus possibly minimizing effects. The only study with a higher ratio (63; ratio of 75/25) assessed inhibitory effects with a flanker task but did not include an experimental assessment conceptually closer to the intervention.

Studies describing effects of IT on drinking behavior have only been conducted in non-clinical samples. Summarizing all existing data, one might suggest cautiously that IT has an effect on drinking behavior during or immediately after the intervention, with three (50, 56, 59) of seven studies reporting such effects and one reporting marginal effects (57). Longer effects on drinking behavior were observed in less than 50% of the studies (see **Figure 2**). Importantly, all of the aforementioned studies were experimental lab studies, thus the majority of their participants might have lacked the motivation to change their drinking behavior, which is discussed to hamper the translation of CBM effects to drinking behavior (24). However, the sole study recruiting participants with a self-reported motivation to change drinking showed no effects of IT on drinking behavior (60).

Although the number of IT studies has nearly doubled since recent meta-analyses (20, 21), one still must acknowledge a considerable variability in size and stability of IT effects on drinking behavior. Furthermore, there is still a lack of clinical studies in patients with AUD and of data from longer follow-up periods, both critical to clinically relevant conclusions. Extending the investigation of IT to clinical samples is also vital because one might tentatively speculate that a pattern similar to ApBM with more promising effects in clinical populations could emerge. A meta-analysis (21) summarizing studies on IT together with other CBMs reported stable effects on bias change but also questioned CBMs effects on drinking behavior. Importantly, our summary of the now larger number of studies on IT does not align with this conclusion: If any, we see an effect of IT on short-term drinking behavior, but not on bias change. This divergent pattern of effects in IT compared to other CBMs might be attributable to a suboptimal assessment of bias in IT: While in AtBM and ApBM studies, most bias assessments used in the studies strongly resembled the intervention to modify the according bias (e.g. VPT to assess attentional bias, VPT-AtBM to modify the bias), this could not be seen in IT as consistently.

### Overall CBM Effects With Respect to Severity of Drinking Behavior

**Figure 4:**
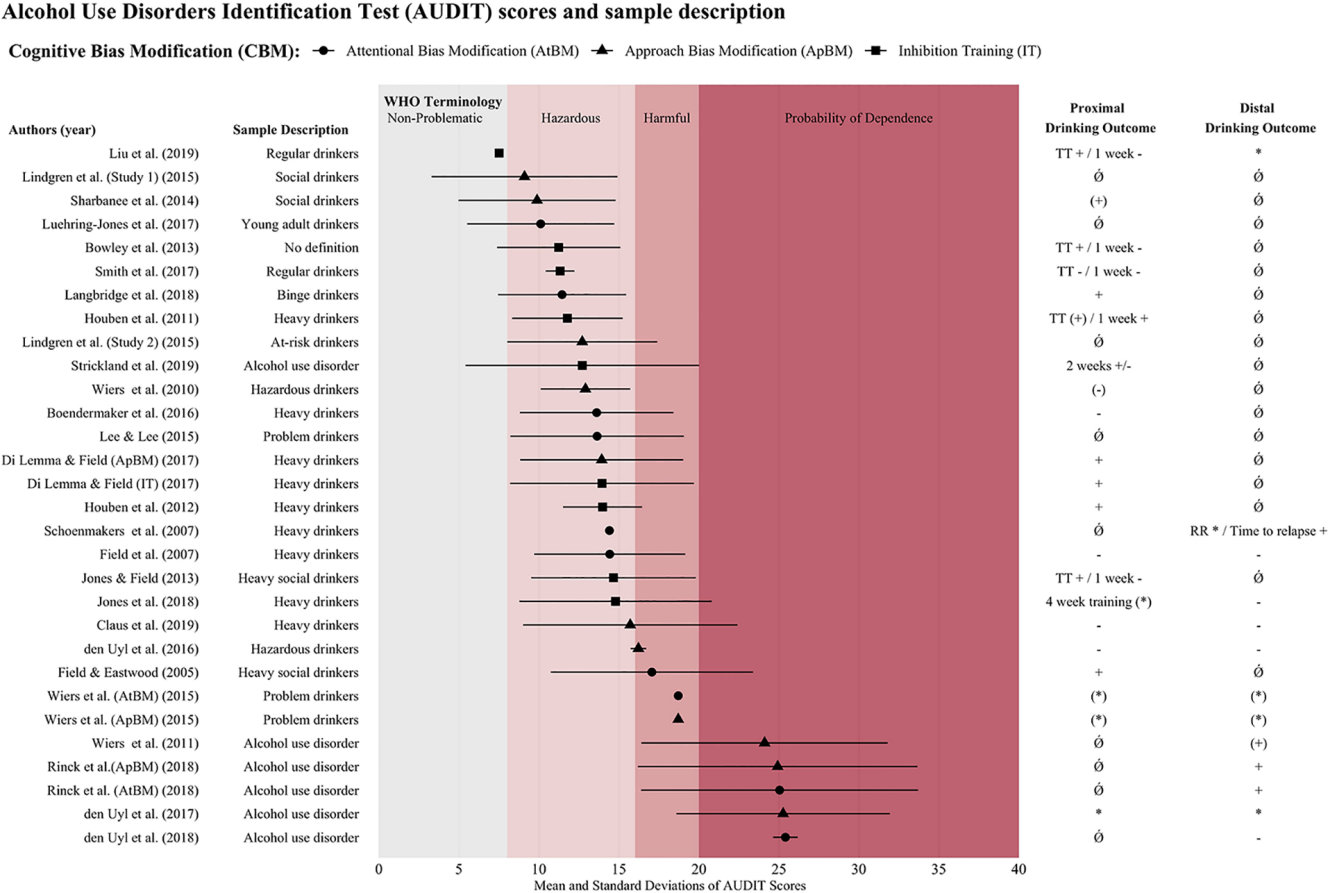
Alignment of studies according to AUDIT means and overview of proximal and distal drinking outcomes. *Notes*. Columns show if study yielded significant effect, indicating that the intervention reduced drinking behavior or relapse rates. Effects are reported as follows + significant effect; (+) trend; +/− mixed results (if different measures were reported); − no effect of the intervention; (−) no effects in ITT analysis, but subgroup with bias change showed drinking reduction; * outcome assessed, but not statistically tested or no control group; (*) no clear effect of intervention, but pre-post reduction in all groups (including control group); Ǿ not measured. Effects were summarized using a conservative approach in the spirit of intention-to-treat analyses (whole group analyses) and only positive effects observed against a control group were seen as clear evidence. Proximal effects: up to 1 month; Distal effects: 1 month and more. Top row: WHO terminology to categorize drinking behavior according to scored AUDIT values: non-problematic: AUDIT 0–7; hazardous: AUDIT 8–15; harmful: AUDIT 16–19; probability of dependence: AUDIT > 20. If not stated in the original research paper, pooled means and standard deviations of AUDIT values were calculated. RR, Relapse rate; TT, Taste test, WHO, World Health Organization.

We set out to compare the effects of CBM with respect to severity of drinking behavior of the investigated sample. As reported, ApBM has shown more consistent beneficial results in clinical samples, while for AtBM, this pattern is less consistent with two positive and two negative trials. Meanwhile, there are no clinical studies on IT in treatment-seeking patients motivated to change drinking In a first-time attempt to analyze the relation between drinking severity and CBM effect over all three interventions, studies using the alcohol use disorder identification test (AUDIT) were aligned according to the mean AUDIT value (**Figure 4**). In a further step, all studies were aligned by sample description with less severe samples at the top and AUD patients at the bottom (**Figure 3**). By doing so, we took a conservative approach, concentrating on those effects reported for the whole group in the spirit of intention-to-treat analyses, while ignoring effects found only in re-analyses focused on a certain subgroup. Furthermore, we considered only the effects that were shown against a control group (i.e. interactions) as clear evidence of a CBM effect. We infer the following from this alignment: There seems to be no continuous improvement of CBM effectiveness with increasing severity, meaning that outcome patterns do not improve with severity within the non-clinical experimental lab studies. Rather, there seems to be a categorical effect with better outcomes in randomized controlled trials with clinical samples compared to experimental lab studies with non-clinical samples. Regarding drinking outcomes, this might be due to the higher motivation to change expected in clinical samples. One must keep in mind however, that this pattern may be due largely to the rather consistent effects that one of the CBM interventions, ApBM, has shown in clinical samples (see **Figure 3**), thus generalization to all CBMs is questionable. Interestingly, the pattern of results within the clinical randomized controlled trials is in line with the proposed working mechanism, which suggests that a change in drinking behavior is driven by the modification of the according bias: **Figure 2** shows that in those RCTs with bias change, drinking behavior changed as well, while those RCTs without bias change showed no drinking behavior changes. Unfortunately, however, this conclusion is supported by only one successful mediation analysis (26), but challenged by two clinical randomized controlled trials that failed to show such mediation effects (27, 28).

What also becomes evident in **Figure 4** is the considerable variation in the use of terminology in sample descriptions: Indeed, the thorough inspection of the literature unveiled a deficiency in standardized definitions for alcohol-related drinking types. When aligning papers along the AUDIT mean values of the investigated samples, it becomes evident that sample descriptions (e.g. HD) are not uniformly used. To our knowledge, no papers have systematically addressed either the problem of drinking type definitions, the variability in terminology and/or the resulting limitations regarding reviews and meta-analyses. One handicap is that some of the terms used for sample description lack a clear, commonly accepted definition (e.g. heavy social drinkers). Furthermore, the alignment with the AUDIT values also shows that the term used may occasionally be misleading or fail to apply to the whole sample, as can be seen from the AUDIT means and standard deviations. For instance, while a sample is called “heavy social drinkers”, this may include AUDIT values indicating hazardous to even alcohol-dependent participants, whereas a sample called “regular drinkers” may include AUDIT values of hazardous drinkers values of hazardous drinkers (as defined e.g. by the World Health Organisation, WHO). In short, only referring to the terminology used in a given publication might imply one drinking type, while the AUDIT scores might point to an additional drinking type. Comparability of studies and clarity of findings would benefit from the use of unified terminology, as has for example been proposed by the WHO (71; see also top of **Figure 4** for WHO terminology: non-problematic use, hazardous use, harmful use, and probability of dependence).

## Summary of Main Findings, Limitations, and Conclusions

Generally, there is still limited research elucidating computerized CBM’s effects. The abundance of cross-sectional studies reporting altered cognitive processing in HD/AUD, which led to the development of computerized interventions, is contrasted by a limited number of longitudinal studies that investigate computerized interventions’ effectiveness, let alone working mechanisms or neuronal effects. We reviewed 40 papers on alcohol-specific computerized CBM interventions. In doing so, we set out to analyze all studies available to date on three alcohol-specific CBMs, namely AtBM, ApBM, and IT, and to summarize this data regarding effects on drinking behavior, experimental tasks, and neurophysiological effects. In contrast to earlier analyses of several (72, 73) or one CBM (18–20,), we summarized data for each CBM separately, subsequently comparing them to one another. Our cautious conclusion after that comparison is as follows: ApBM has shown the most consistent effects in clinical samples, while evidence on AtBM is more inconsistent, and data on IT is still lacking important information, such as the effectiveness in clinical samples and the effects on inhibitory control itself.

Several limitations when comparing CBM studies and in terms of this review should be addressed. Firstly, despite having carefully searched several databases and articles, we cannot fully rule out having missed a paper that should have been included in this review. Secondly, because the non-uniform use of terminology regarding the subjects’ drinking behavior complicates conclusions with respect to severity, we strongly encourage future studies to lean on the WHO’s definitions depending on the achieved AUDIT score across the sample (71). Furthermore, the studies in this review primarily included student samples. Only few non-clinical studies investigated representative and socio-economically mingled samples, and less than one-third of the available studies were conducted in clinical populations, in which these interventions should ultimately be implemented. Beside the fact that students generally represent a rather young and cognitively strong sample, which may impact results, students may also lack the motivation to change their drinking behavior. Motivation to change, which is discussed as a prerequisite or mediator for a successful intervention (e.g. 17, 24), is assessed in some studies only (32, 34, 41, 49), and even more seldom included in the analyses (see **Supplementary Table 1**). It is highly probable that on average, patients show a higher motivation to change their drinking behavior when compared to non-clinical samples. But even among patient samples, motivation to change is not a fully stable and omnipresent phenomenon. Thus, future research would profit from the assessment of motivation to change in the participants. Also, baseline levels of the biases which are to be targeted by the intervention may vary among individuals. Unfortunately, those levels are rarely reported, even if they have been shown to moderate an intervention’s effectiveness (26, but see 27) and were considered relevant in a recent review on AtBM (75).

Further, regarding methodological variation, the number of sessions ranged between one and 14 in the articles reviewed, and within certain studies, participants completed different numbers of interventions. Earlier research suggests that it is unlikely for one session to have a therapeutic effect (21). For ApBM, it has been demonstrated that the optimal number of training sessions shows strong interindividual variance, with six sessions being the mean (76). This suggests that most of the studies reviewed here may not have reached the optimal effect with the intervention. In addition, the web-based vs. laboratory setting may have led to differences in study compliance. An alternative explanation for the discrepant results regarding AtBM might be attributable to the fact that the three studies testing multiple sessions in HD conducted the training sessions at home (i.e. over the web), which might have reduced the participants compliance, attention, and/or motivation (24, 74). Also, most papers did not tailor the pictures presented according to participants’ drinks of choice. Since a subjects’ preferred drink has a higher incentive value than other types of beverage, the use of individually meaningful stimulus material may play a pivotal role and influence the effects of CBM interventions. Lastly, the lack of neurophysiological assessments (see **Table 1**) investigating neuronal processes underlying bias change and effects on drinking outcomes is regrettable and hampers any firm conclusions in this realm. As all three of the CBMs can at least in part be linked to basic and clinical neuroscientific concepts, the scientific anchoring of these interventions would profit from expanded knowledge regarding their neuroscientific effects.

Overall, it seems advisable for future research to concentrate on clinical populations, include the assessment of motivation and baseline bias levels, analyze mediation, and neurophysiological effects and use longer follow-up periods to augment the understanding of computerized interventions’ effects.

## Author Contributions

HB conducted the literature search in January and May 2019. HB and MS equally contributed to tables, figures, and writing the initial manuscript. HB, MS, and FM elaborated conclusions. HB, MS, RT, LS, and FM contributed to revising the manuscript and finalizing it. HB and FM assessed each study for quality criteria. Whenever unclear, MS was asked for her opinion.

## Funding

This research was supported by a grant from the Swiss National Science Foundation (grant-nr: 105319_159286) to MS, LS and FM.

## Conflict of Interest

The authors declare that the research was conducted in the absence of any commercial or financial relationships that could be construed as a potential conflict of interest.

## References

[1] Rehm J The risks associated with alcohol use and alcoholism. Alcohol Res Health (2011) 34(2):135–43. PMC3307043 22330211

[2] Anton RF O’Malley SS Ciraulo DA Cisler RA Couper D Donovan DM Combined pharmacotherapies and behavioral interventions for alcohol dependence: the COMBINE study: a randomized controlled trial. JAMA (2006) 295(17):2003–17. 10.1001/archpsyc.65.2.135 16670409

[3] Wiers RW Becker D Holland RW Moggi F Lejuez CW Addictions. A Social Psychological Perspective. In: Kopetz CE Lejuez CWeditors. Cognitive motivational processes underlying addiction treatment. Routledge (2016). p. 201–36.

[4] Franken IH Rosso M Van Honk J Selective memory for alcohol cues in alcoholics and its relation to craving. Cogn Ther Res (2003) 27(4):481–8. 10.1023/A:1025480615623

[5] Field M Cox WM Attentional bias in addictive behaviors: a review of its development, causes, and consequences. Drug Alcohol Depend (2008) 97(1):1–20. 10.1016/j.drugalcdep.2008.03.030 18479844

[6] Smith JL Mattick RP Jamadar SD Iredale JM Deficits in behavioural inhibition in substance abuse and addiction: a meta-analysis. Drug Alcohol Depend (2014) 145:1–33. 10.1016/j.drugalcdep.2014.08.009 25195081

[7] Field M Wiers RW Christiansen P Fillmore MT Verster JC Acute alcohol effects on inhibitory control and implicit cognition: implications for loss of control over drinking. Alcohol Clin Exp Res (2010) 34(8):1346–52. 10.1111/j.1530-0277.2010.01218.x PMC2999764 20491732

[8] Stein M Fey W Koenig T Oehy J Moggi F context-specific inhibition is related to craving in alcohol use disorders: a dangerous imbalance. Alcohol Clin Exp Res (2018) 42(1):69–80. 10.1111/acer.13532 29044574

[9] Volkow ND Baler R Addiction science: uncovering neurobiological complexity. Neuropharmacology (2014) 76:235–49. 10.1016/j.neuropharm.2013.05.007 PMC3818510 23688927

[10] Field M Duka T Eastwood B Child R Santarcangelo M Gayton M Experimental manipulation of attentional biases in heavy drinkers: do the effects generalise? Psychopharmacol (Berl) (2007) 192(4):593–608. 10.1007/s00213-007-0760-9 17361393

[11] Palfai TP Ostafin BD Alcohol-related motivational tendencies in hazardous drinkers: assessing implicit response tendencies using the modified-IAT. Behav Res Ther (2003) 41(10):1149–62. 10.1016/S0005-7967(03)00018-4 12971937

[12] Field M Marhe R Franken IHA The clinical relevance of attentional bias in substance use disorders. CNS Spectrums (2014) 19(3):225–30. 10.1017/S1092852913000321 23663386

[13] Cox WM Fadardi JS Pothos EM The addiction-stroop test: theoretical considerations and procedural recommendations. Psychol Bull (2006) 132(3):443. 10.1037/0033-2909.132.3.443 16719569

[14] Watson P De Wit S Hommel B Wiers RW Motivational mechanisms and outcome expectancies underlying the approach bias toward addictive substances. Front Psychol (2012) 3:440. 10.3389/fpsyg.2012.00440 23133434 PMC3490330

[15] Jones A Christiansen P Nederkoorn C Houben K Field M Fluctuating disinhibition: implications for the understanding and treatment of alcohol and other substance use disorders. Front Psychiatry (2013) 4:140. 10.3389/fpsyt.2013.00140 24155728 PMC3804868

[16] Lindenmeyer J Is there a case in neuropsychological relapse prevention in alcoholic patients? Suchttherapie (2010) 11(04):166–72.

[17] Gladwin TE Wiers CE Wiers RW Interventions aimed at automatic processes in addiction: considering necessary conditions for efficacy. Curr Opin Behav Sci (2017) 13:19–24. 10.1016/j.cobeha.2016.08.001

[18] Kakoschke N Kemps E Tiggemann M Approach bias modification training and consumption: A review of the literature. Addict Behav (2017) 64:21–8. 10.1016/j.addbeh.2016.08.007 27538198

[19] Christiansen P Schoenmakers TM Field M Less than meets the eye: reappraising the clinical relevance of attentional bias in addiction. Addict Behav (2015) 44:43–50. 10.1016/j.addbeh.2014.10.005 25453782

[20] Allom V Mullan B Hagger M Does inhibitory control training improve health behaviour? A meta-analysis. Health Psychol Rev (2016) 10(2):168–86. 10.1080/17437199.2015.1051078 26058688

[21] Cristea IA Kok RN Cuijpers P The effectiveness of cognitive bias modification interventions for substance addictions: a meta-analysis. PloS One (2016) 11(9):e0162226. 10.1371/journal.pone.0162226 27611692 PMC5017662

[22] Stock A-K Barking up the Wrong Tree: Why and How We May Need to Revise Alcohol Addiction Therapy. Front Psychol (2017) 8:884. 28611718 10.3389/fpsyg.2017.00884 PMC5447061

[23] Jones A Di Lemma LC Robinson E Christiansen P Nolan S Tudur-Smith C Inhibitory control training for appetitive behaviour change: A meta-analytic investigation of mechanisms of action and moderators of effectiveness. Appetite (2016) 97:16–28. 10.1016/j.appet.2015.11.013 26592707

[24] Wiers RW Boffo M Field M What’s in a Trial? On the importance of distinguishing between experimental lab studies and randomized controlled trials: the case of cognitive bias modification and alcohol use Disorders. J Stud Alcohol Drugs (2018) 79(3):333–43. 10.15288/jsad.2018.79.333 29885138

[25] DiClemente CC Bellino LE Neavins TM Motivation for change and alcoholism treatment. Alcohol Res Health (1999) 23(2):87–92. PMC6760428 10890801

[26] Eberl C Wiers RW Pawelczack S Rinck M Becker ES Lindenmeyer J Approach bias modification in alcohol dependence: do clinical effects replicate and for whom does it work best? Dev Cognit Neurosci (2013) 4:38–51. 10.1016/j.dcn.2012.11.002 23218805 PMC6987692

[27] Rinck M Wiers RW Becker ES Lindenmeyer J Relapse prevention in abstinent alcoholics by cognitive bias modification: clinical effects of combining approach bias modification and attention bias modification. J Consult Clin Psychol (2018) 86(12):1005–16. 10.1037/ccp0000321 30507226

[28] Wiers RW Eberl C Rinck M Becker ES Lindenmeyer J Retraining automatic action tendencies changes alcoholic patients’ approach bias for alcohol and improves treatment outcome. Psychol Sci (2011) 22(4):490–7. 10.1177/0956797611400615 21389338

[29] Clerkin EM Magee JC Wells TT Beard C Barnett NP Randomized controlled trial of attention bias modification in a racially diverse, socially anxious, alcohol dependent sample. Behav Res Ther (2016) 87:58–69. 10.1016/j.brat.2016.08.010 27591918 PMC5127758

[30] den Uyl TE Gladwin TE Lindenmeyer J Wiers RW A clinical trial with combined transcranial direct current stimulation and attentional bias modification in alcohol-dependent patients. Alcohol Clin Exp Res (2018) 42(10):1961–9. 10.1111/acer.13841 PMC6175348 30025152

[31] Schoenmakers TM de Bruin M Lux IF Goertz AG Van Kerkhof DH Wiers RW Clinical effectiveness of attentional bias modification training in abstinent alcoholic patients. Drug Alcohol Depend (2010) 109(1-3):30–6. 10.1016/j.drugalcdep.2009.11.022 20064698

[32] Boendermaker WJ Sanchez Maceiras S Boffo M Wiers RW Attentional bias modification with serious game elements: evaluating the shots game. JMIR Serious Games (2016) 4(2):e20. 10.2196/games.6464 27923780 PMC5174726

[33] Cox WM Fadardi JS Hosier SG Pothos EM Differential effects and temporal course of attentional and motivational training on excessive drinking. Exp Clin Psychopharmacol (2015) 23(6):445–54. 10.1037/pha0000038 PMC4655870 26348159

[34] Fadardi JS Cox WM Reversing the sequence: reducing alcohol consumption by overcoming alcohol attentional bias. Drug Alcohol Depend (2009) 101(3):137–45. 10.1016/j.drugalcdep.2008.11.015 19193499

[35] Field M Eastwood B Experimental manipulation of attentional bias increases the motivation to drink alcohol. Psychopharmacol (Berl) (2005) 183(3):350–7. 10.1007/s00213-005-0202-5 16235080

[36] Langbridge JE Jones RD Canales JJ A neurophysiological and behavioral assessment of interventions targeting attention bias and sense of control in binge drinking. Front Hum Neurosci (2018) 12:538. 10.3389/fnhum.2018.00538 30687051 PMC6337047

[37] Lee S Lee J-H The effect of automatic attentional bias modification on alcohol ambivalence. Addictive Behav (2015), 46: 58–64. 10.1016/j.addbeh.2015.03.01025800362 25800362

[38] Luehring-Jones P Louis C Dennis-Tiwary TA Erblich J A single session of attentional bias modification reduces alcohol craving and implicit measures of alcohol bias in young adult drinkers. Alcohol Clin Exp Res (2017) 41(12): 2207–16. 10.1111/acer.13520 PMC5711540 28992377

[39] McGeary JE Meadows SP Amir N Gibb BE Computer-delivered, home-based, attentional retraining reduces drinking behavior in heavy drinkers. Psychol Addict Behav (2014) 28(2):559–62. 10.1037/a0036086 PMC4068274 24955674

[40] Schoenmakers T Wiers RW Jones BT Bruce G Jansen AT Attentional re-training decreases attentional bias in heavy drinkers without generalization. Addiction (2007) 102(3):399–405. 10.1111/j.1360-0443.2006.01718.x 17298647

[41] Wiers RW Houben K Fadardi JS van Beek P Rhemtulla M Cox WM Alcohol cognitive bias modification training for problem drinkers over the web. Addict Behav (2015c) 40:21–6. 10.1016/j.addbeh.2014.08.010 25218067

[42] den Uyl TE Gladwin TE Rinck M Lindenmeyer J Wiers RW A clinical trial with combined transcranial direct current stimulation and alcohol approach bias retraining. Addict Biol (2017) 22(6):1632–40. 10.1111/adb.12463 27790791

[43] Loijen A Rinck M Walvoort SJW Kessels RPC Becker ES Egger JIM Modification of automatic alcohol-approach tendencies in alcohol-dependent patients with mild or major neurocognitive disorder. Alcohol Clin Exp Res (2018) 42(1):153–61. 10.1111/acer.13529 29034489

[44] Manning V Staiger PK Hall K Garfield JB Flaks G Leung D Cognitive bias modification training during inpatient alcohol detoxification reduces early relapse: a randomized controlled trial. Alcohol Clin Exp Res (2016) 40(9):2011–9. 10.1111/acer.13163 27488392

[45] Gladwin TE Rinck M Eberl C Becker ES Lindenmeyer J Wiers RW Mediation of cognitive bias modification for alcohol addiction via stimulus-specific alcohol avoidance association. Alcohol Clin Exp Res (2015) 39(1):101–7. 10.1111/acer.12602 25623410

[46] Wiers CE Stelzel C Gladwin TE Park SQ Pawelczack S Gawron CK Effects of cognitive bias modification training on neural alcohol cue reactivity in alcohol dependence. Am J Psychiatry (2015b) 172(4):335–43. 10.1176/appi.ajp.2014.13111495 25526597

[47] Wiers CE Ludwig VU Gladwin TE Park SQ Heinz A Wiers RW Effects of cognitive bias modification training on neural signatures of alcohol approach tendencies in male alcohol-dependent patients. Addict Biol (2015a) 20(5):990–9. 10.1111/adb.12221 25639749

[48] Claus ED Klimaj SD Chavez R Martinez AD Clark VP A randomized trial of combined tDCS over right inferior frontal cortex and cognitive bias modification: Null effects on drinking and alcohol approach bias. Alcohol Clin Exp Res (2019) 43(7):1591–9. 10.1111/acer.14111 PMC6602850 31081924

[49] den Uyl TE Gladwin TE Wiers RW Electrophysiological and behavioral effects of combined transcranial direct current stimulation and alcohol approach bias retraining in hazardous drinkers. Alcohol Clin Exp Res (2016) 40(10):2124–33. 10.1111/acer.13171 27558788

[50] Di Lemma LCG Field M Cue avoidance training and inhibitory control training for the reduction of alcohol consumption: a comparison of effectiveness and investigation of their mechanisms of action. Psychopharmacol (Berl) (2017) 234(16):2489–98. 10.1007/s00213-017-4639-0 PMC5537323 28551714

[51] Hahn AM Simons RM Simons JS Wiers RW Welker LE Can cognitive bias modification simultaneously target two behaviors? Approach bias retraining for alcohol and condom use. Clin Psychol Sci (2019). 7(5):1078–93. 10.1177/2167702619834570 PMC6936737 31890350

[52] Leeman RF Nogueira C Wiers RW Cousijn J Serafini K DeMartini KS A test of multisession automatic action tendency retraining to reduce alcohol consumption among young adults in the context of a human laboratory paradigm. Alcoholism-Clin Exp Res (2018) 42(4):803–14. 10.1111/acer.13613 PMC5995797 29450895

[53] Lindgren KP Wiers RW Teachman BA Gasser ML Westgate EC Cousijn J Attempted training of alcohol approach and drinking identity associations in us undergraduate drinkers: null results from two studies. PloS One (2015) 10(8):e0134642. 10.1371/journal.pone.0134642 26241316 PMC4524630

[54] Sharbanee JM Hu L Stritzke WGK Wiers RW Rinck M MacLeod C The effect of approach/avoidance training on alcohol consumption is mediated by change in alcohol action tendency. PloS One (2014) 9(1):e85855. 10.1371/journal.pone.0085855 24465750 PMC3899082

[55] Wiers RW Rinck M Kordts R Houben K Strack F Retraining automatic action-tendencies to approach alcohol in hazardous drinkers. Addiction (2010) 105(2):279–87. 10.1111/j.1360-0443.2009.02775 20078486

[56] Bowley CFC Hegarty B Johnstone S Smith J Kelly P Rushby J The effects of inhibitory control training on alcohol consumption, implicit alcohol-related cognitions and brain electrical activity. Int J Psychophysiol (2013) 89(3):342–8. 10.1016/j.ijpsycho.2013.04.011 23623953

[57] Houben KNC Wiers RW Jansen A Resisting temptation: decreasing alcohol-related affect and drinking behavior by training response inhibition. Drug Alcohol Depend (2011) 116(1-3):132–6. 10.1016/j.drugalcdep.2010.12.011 21288663

[58] Houben KHRC Nederkoorn C Jansen A Beer a no-go: learning to stop responding to alcohol cues reduces alcohol intake via reduced affective associations rather than increased response inhibition. Addiction (2012) 107(7):1280–7. 10.1111/j.1360-0443.2012.03827.x 22296168

[59] Jones A Field M The effects of cue-specific inhibition training on alcohol consumption in heavy social drinkers. Exp Clin Psychopharmacol (2013) 21(1):8. 10.1037/a0030683 23181512

[60] Jones A McGrath E Robinson E Houben K Nederkoorn C Field M A randomized controlled trial of inhibitory control training for the reduction of alcohol consumption in problem drinkers. J Consulting Clin Psychol (2018) 86(12):991–1004. 10.1037/ccp0000312 PMC6277130 30507225

[61] Kilwein TM Bernhardt KA Stryker ML Looby A Decreased alcohol consumption after pairing alcohol-related cues with an inhibitory response. J Subst Use (2018) 23(2): 154–61. 10.1080/14659891.2017.1378736

[62] Liu Q Hu L Smith JL Mewton LR Can inhibitory training produce reductions in drinking? Evaluating the influence of the control condition. J Stud Alcohol Drugs (2019) 80(1):96–101. 10.15288/jsad.2019.80.96 30807280

[63] Smith JL Dash NJ Johnstone SJ Houben K Field M Current forms of inhibitory training produce no greater reduction in drinking than simple assessment: A preliminary study. Drug Alcohol Depend (2017) 173:47–58. 10.1016/j.drugalcdep.2016.12.018 28196787

[64] Strickland JC Hill JC Stoops WW Rush CR Feasibility, acceptability, and initial efficacy of delivering alcohol use cognitive interventions via crowdsourcing. Alcoholism-Clin Exp Res (2019) 43(5):888–99. 10.1111/acer.13987 30888705

[65] Higgins JP Altman DG Gøtzsche PC Jüni P Moher D Oxman AD The Cochrane Collaboration’s tool for assessing risk of bias in randomised trials. BMJ (2011) 343:d5928. 10.1136/bmj.d5928 22008217 PMC3196245

[66] Cristea IA Kok RN Cuijpers P Persistent Double Standards in Evaluating the Effectiveness of Cognitive Bias Modification: A Commentary on Wiers (2018). J Stud Alcohol Drugs (2018) 79(3):344–5. 10.15288/jsad.2018.79.344 29885139

[67] Verdejo-Garcia A Cognitive training for substance use disorders: neuroscientific mechanisms. Neurosci Biobehav Rev (2016) 68:270–81. 10.1016/j.neubiorev.2016.05.018 27236041

[68] Schachar R Logan GD Robaey P Chen S Ickowicz A Barr C Restraint and cancellation: multiple inhibition deficits in attention deficit hyperactivity disorder. J Abnormal Child Psychol (2007) 35(2):229–38. 10.1007/s10802-006-9075-2 17351752

[69] Verbruggen F Logan GD Automatic and controlled response inhibition: associative learning in the go/no-go and stop-signal paradigms. J Exp Psychol: Gen (2008) 137(4):649. 10.1037/a0013170 18999358 PMC2597400

[70] Veling H Holland RW van Knippenberg A When approach motivation and behavioral inhibition collide: behavior regulation through stimulus devaluation. J Exp Soc Psychol (2008) 44(4):1013–9. 10.1016/j.jesp.2008.03.004

[71] Babor TF Higgins-Biddle J Saunders J Monteiro M (2001). The alcohol use disorders identification test (AUDIT). Guidelines for use in primary care.

[72] Gladwin TE Wiers CE Wiers RW Cognitive neuroscience of cognitive retraining for addiction medicine: from mediating mechanisms to questions of efficacy. Prog Brain Res (2016) 224:323–44. 10.1016/bs.pbr.2015.07.021 26822365

[73] Copersino ML Cognitive Mechanisms and Therapeutic Targets of Addiction. Curr Opin Behav Sci (2017) 13:91–8. 10.1016/j.cobeha.2016.11.005 PMC5461927 28603756

[74] Mogoaşe C David D Koster EH Clinical efficacy of attentional bias modification procedures: an updated meta-analysis. J Clin Psychol (2014) 70(12):1133–57. 10.1002/jclp.22081 24652823

[75] Heitmann J Bennik EC van Hemel-Ruiter ME de Jong PJ The effectiveness of attentional bias modification for substance use disorder symptoms in adults: a systematic review. Systematic Rev (2018) 7(1):160. 10.1186/s13643-018-0822-6 PMC6186103 30316302

[76] Eberl C Wiers RW Pawelczack S Rinck M Becker ES Lindenmeyer J Implementation of approach bias re-training in alcoholism-how many sessions are needed? Alcohol Clin Exp Res (2014) 38(2):587–94. 10.1111/acer.12281 24164417

