# Supplementary material for: Alcohol-Specific Computerized Interventions to Alter Cognitive Biases: A Systematic Review of Effects on Experimental Tasks, Drinking Behavior, and Neuronal Activation
Source: Front Psychiatry. 2020 Jan 9;10:871. doi: 10.3389/fpsyt.2019.00871 (PMC6970199; doi:10.3389/fpsyt.2019.00871)
Supplement: Supplementary file 1 [file Table_1.docx]

Supplementary table 1: Judgment of risk of bias based on criteria included in the Cochrane Collaboration’s Risk of Bias guidelines and other methodological factors of included studies

| **Supplementary material table 1A: Attentional Bias Modification (AtBM) studies and risk of bias of single studies** | | | | | | | |  |  |
| --- | --- | --- | --- | --- | --- | --- | --- | --- | --- |
| **Author(s),  year** | **Sample characteristics** | **# of sessions /**  **setting** | **Motivation assessed /  included in analyses** | **Power analysis / N achieved?** | **Randomization**  **bias** | **Blinding**  **bias** | **Attrition bias: Initial N / N for analyses** | |  |
| **RCT** |  |  |  |  |  |  |  | |  |
| Clerkin et  al., 2016 | AUD  Clinical sample  M(a) 44.3 (10.9)  41% females | 8  Lab | Not assessed | No / - | Low | Low: Double-blind | Low  94/ 86 | |  |
| den Uyl, Gladwin, Lindenmeyer et al., 2018 | AUD  Clinical sample  M(a) 48.6 (0.9)  25% females | 4  Lab | Not assessed | No / - | Low | Low: Double-blind | Low  98 / 83 | |  |
| Rinck et  al., 2018 | AUD  Clinical sample  M(a) 45.8 (9.5)  27% females | 6  Lab (clinic) | Not assessed | Yes / No | Low | High | Low, High  1405 / 1108 /  774 / 705 | |  |
| Schoenmakers et  al., 2010 | AUD  Clinical sample M(a) 45.0 (9.9)  23% females | 5  Lab (clinic) | Not assessed | No / - | Low | Low: Double-blind | Low  43 / 35 / 31 | |  |
|  |  |  |  |  |  |  |  | |  |
| **ELS** |  |  |  |  |  |  |  | |  |
| Boendermaker et  al., 2016 | HD  Students M(a) 21.2 (1.8)  (range 18-28)  71 % females | 4  Lab | Short version of the Readiness to Change Questionnaire (RCQ) / Analysis of motivation change over time | No / - | Low | Unclear | Low  96 / 94 / 90 | |  |
| Cox et al., 2015 | Harmful drinkers  General population M(a) 28.8 (14.4)  52% females | 4  Lab | Readiness to change Questionnaire (RCQ) / Baseline differences between groups | No / - | Unclear | High: Single-blind | Low, High  148 / 117 / 74 | |  |
| Fadardi &  Cox, 2009 | SD/HZD/HFD  General population  M(a) 32.6 (11.1)  48% females | 0 - 4  Lab | Readiness to Change Questionnaire (RTCQ) / Analysis of motivation change over time | No / - | (no randomi-  zation) | Unclear | Low  221 / 200 | |  |
| Field & Eastwood, 2005 | Heavy social  drinkers  General population  M(a) 22.1 (3.9)  50% females | 1  Lab | Not assessed | No / - | Low | Unclear | Low  40 / 40 | |  |
| Field et  al., 2007 | HD  General population  M(a) 23.1 (8.7) 66% females | 1  Lab | Not assessed | No / - | Low | Low: Double-blind | Low  60 / 59 /  59 / 59 | |  |
| Langbridge  et al., 2018 | Binge drinkers  General population  Median age across all groups: 22 (aged 16-50)  56% females | 1  Lab | Not assessed | Yes / Yes | Unclear | Unclear | Low  51 / 50 | |  |
| Lee & Lee,  2015 | Problem drinkers  Students  M(a) 22.0 (2.6)  61% females | 1  Lab | Readiness to Change Questionnaire (RTCQ) / Analysis of motivation change over time. | No / - | Low | Low: Double-blind | Low  48 / 43 | |  |
| Luehring-Jones  et al., 2017 | Young adult drinkers  General population  M(a) 22.0 (2.2)  55% females | 1  Lab | Not assessed | No / - | Low | High: Single-blind | Low  60 / 49-52 | | |
| McGeary et  al., 2014 | HD  Students M(a) 19.0 (1.1)  0% females | 8  Web-based  (home) | Not assessed | No / - | Low | High: Single-blind | Low  41 / 39 | |  |
| Schoenmakers  et al., 2007 | HD  Students  M(a) 21.4 (2.0)  0% females | 1  Lab | Not assessed | No / - | Low | Unclear | Low  106 / 105 / 103 | |  |
| Wiers, Houben  et al., 2015 | Problem drinkers  General population  M(a) 47.4 (no SD)  42.5% females | 4  Web-based  (home) | Readiness to Change Questionnaire (RTCQ) / No analysis. | No / - | Low | Unclear | Low  136 / 132 | |  |

| **Supplementary material table 1B: Approach Bias Modification (ApBM) studies and risk of bias of single studies** | | | | | | | |
| --- | --- | --- | --- | --- | --- | --- | --- |
| **Author(s),  year** | **Sample characteristics** | **# of  sessions / setting** | **Motivation assessed / included in analyses** | **Power analysis / N achieved?** | **Randomization**  **bias** | **Blinding**  **bias** | **Attrition bias: Initial N / N for analyses** |
| **RCT** |  |  |  |  |  |  |  |
| den Uyl, Gladwin, Rinck et al., 2017 | AUD  Clinical sample M(a) 47.0 (8.8)  33% females | 4  Lab  (clinic) | Not assessed | No / - | Low | Low: Double-  blind | Low  100 / 91 |
| Eberl et  al., 2013 | AUD  Clinical sample M(a) 46.0 (9.0)  Sex ratio not reported | 12  Lab  (clinic) | Not assessed | No / - | Low | Unclear | Low  509 / 499 /  475 |
| Loijen et  al., 2017 | AUD with alcohol-induced neuro-cognitive disorders  Clinical sample  M(a) 51.9 (15.6)  25% females | 6  Lab  (clinic) | Not assessed | No / - | Unclear | Unclear | Low  106 / 85 |
| Manning et  al., 2016 | AUD  Clinical sample  M(a) 40.0  49.4% females | 4  Lab  (clinic?) | Not assessed | Yes / Yes | Low | Unclear | Low  87 / 83 / 71 |
| Rinck et  al., 2018 | AUD  Clinical sample  M(a) 45.7 (9.4) 26.6% females | 6  Lab  (clinic) | Not assessed | Yes / No | Low | High | Low, High  1405 / 1108 /  773 /705 |
| Wiers, Eberl  et al., 2011 | AUD  Clinical sample  M(a) 45.3 (8.0)  24% females | 4  Lab  (clinic) | Not assessed | No / - | Low | High | Low  214 / 173 / 181 |
| Gladwin et al., 2015  (re-analysis of Wiers, Eberl et al. (2011) | AUD  Clinical sample M(a) 45.3 (8.0)  24% females | 4  Lab  (clinic) | Not assessed | No / - | Low | High | Low  214 / 184 |
| Wiers, Stelzel  et al., 2015 | AUD  Clinical sample  M(a) 44.0 (7.6)  0% females | 6  Lab | Not assessed | No / - | Low | Low: Double-  blind | Low  36 / 32 |
| Wiers, Ludwig, et  al., 2015 (sample of Wiers, Stelzel et al. (2015)) | AUD Clinical sample  M(a) 43.9  0% females | 6  Lab | Not assessed | No / - | Low | Low: Double-  blind | Low  36 / 26 |
|  |  |  |  |  |  |  |  |
|  |  |  |  |  |  |  |  |
|  |  |  |  |  |  |  |  |
| **ELS** |  |  |  |  |  |  |  |
| Claus et  al., 2019 | HD  Students  M(a) 24.5 (2.7)  31.7% females | 4  Lab | Not assessed | Yes / No | Low | Low: Double-  blind | Low  91 / 79 / 70 |
| den Uyl, Gladwin, and Wiers, 2016 | HZD  Students M(a) 21.8 (3.2)  65.4% females | 3  Lab | Readiness to change questionnaire (RTCQ) / Baseline differences between groups | No / - | Low | Low: Double-  blind | Low  86/ 78 |
| Di Lemma &  Field, 2017 | HD  General population  M(a) 20.4 (2.1)  71.7% females | 1  Lab | Readiness to change questionnaire (RTCQ) / Baseline differences between groups | No / - | Low | High: Single-  blind | Low  120/ 119 |
| Hahn et  al., 2019 | High risk young adults  Students M(a) 20.0 (1.5)  Approximately  66% females | 4  Lab | Not assessed | Yes / Yes | Low | Unclear | Low  102 / 91 / 85 |
| Leemann et  al., 2018 | HD  General Population  21 – 25 years old  46.4% females | 4  Lab | Not assessed | Yes / Yes | Low | Unclear | Low  72 / 69 / 68 |
| Lindgren  et al., 2015 | Study 1: social drinkers  Students  M(a) 20.5 (1.4)  54.6% females  Study 2: At-risk drinkers  Students  M(a) 20.5 (2.1)  52.1% females | 2  Lab | Not assessed | No / - | Low | Unclear | Study 1: Low  295 / 224  Study 2: Low  288 / 236 |
| Sharbanee  et al., 2014 | Social drinkers  Students  M(a) 19.4 (2.1)  66.2% females | 1  Lab | Stages of Change Readiness and Treatment Eagerness Scale (SOCRATES) / Baseline differences between groups | No / - | Unclear | Unclear | Low  74 / 74 |
| Wiers, Houben  et al., 2015 | Problem  drinkers  General population  M(a) 48.3 (no SD)  42.5% females | 4  Web-based  (home) | Readiness to Change Questionnaire (RCQ). No analysis | No / - | Low | Unclear | High  314 / 136 / 109 /  87 |
| Wiers, Rinck  et al., 2010 | HZD  Students aged 18-28  0% females | 1  Lab | Not assessed | No / - | Low | High: Single-  blind | Low  42 / 41 |

| **Supplementary material table 1C: Inhibition Training (IT) studies and risk of bias of single studies** | | | | | | | |
| --- | --- | --- | --- | --- | --- | --- | --- |
| **Author(s),  year** | **Sample**  **characteristics** | **# of**  **sessions / setting** | **Motivation assessed / included in analyses** | **Power analysis /**  **N achieved?** | **Randomization**  **bias** | **Blinding**  **bias** | **Attrition bias: Initial N / N for analyses** |
| **ELS** |  |  |  |  |  |  |  |
| Bowley et  al., 2013 | (no definition)  Students  M(a) 20.8 (2.0)  23.6% females | 1  Lab | Not assessed | No / - | Low | Unclear | Low  66 / 59 |
| Di Lemma &  Field, 2017 | HD  General population  M(a) 20.3 (2.0)  71.7% females | 1 (+ 2 times  booster training trials)  Lab | Readiness to change questionnaire (RTCQ) / Baseline differences between groups | No / - | Low | High: Single-  blind | Low  120 / 119 |
| Houben et  al., 2011 | HD  Students  M(a) 22.4 (4.9) 63.5% females | 1  Lab | Not assessed | No / - | Low | Unclear | Low  52 / 51 |
| Houben et  al., 2012 | HD  Students M(a) 20.9 (1.8)  42.1% females | 1  Lab | Not assessed | Yes / Yes | Low | Unclear | Low  57 / 56 |
| Jones &  Field, 2013 | Heavy social drinkers  General population M(a) 20.8 (2.7)  54.4% females | 1  Lab | Not assessed | No / - | Low | Unclear | Low  90 / 90 |
| Jones et  al., 2018 | HD  General population  M(a) 41.3 (11.7)  47.2% females | 8-14  Web-based  (home) | Assessed at post-intervention / no analysis | Yes / Unclear | Low | Low: Double-  blind | Low, High  229 / 205 / 152 /  140 / 112 |
| Kilwein et  al., 2017 | HD  General population  M(a) 22.6 (2.1)  0% females | 1  Lab | Not assessed | No / - | Low | High: Single-  blind | Low  47 / 44 |
| Liu et al.,  2019 | Regular drinkers  Students  Aged 18-30  65% females | 1  Lab | Not assessed | No / - | Low | High: Single-  blind | Low  88 / 81 |
| Smith et  al., 2017 | Regular drinkers  General population  M(a) 21.7 (0.7)  36.8% females | 1  Lab | Not assessed | No / - | Low | High: Single-  blind | Low  114 / 110 |
| Strickland et  al., 2019 | AUD  General population  M(a): 34.3 (9.7)  51.1.% females | 14  Web-based  (home) | Not assessed | Unclear | Low | Unclear | Low  476 / 402 |

*Notes.* Whenever possible, the risk of bias according to the Cochrane Collaboration’s Risk of Bias guidelines was judged to be high, low, or unclear, with a low risk of bias being the most optimal (last three columns). Description of columns from left to right: First author(s) and year of publication; Sample composition (e.g., student sample, general population, or clinical sample; mean age (+ standard deviation) of complete sample; sex ratio (% females)); number of intervention sessions and setting (lab or web-based (i.e., home)); if motivation to change behavior was assessed and included in analyses; if power analysis was conducted prior to study onset and if so, if calculated N was achieved; randomization bias: randomized allocation to conditions; blinding bias: blinding procedure (single- or double-blind); attrition bias (initial sample size at randomization, and sample size for (sub)analyses). Abbreviations: AUD = individuals with Alcohol Use Disorders; HD = heavy drinkers; HFD = harmful drinkers; HZD = hazardous drinkers; N = number of subjects; M(a) = mean age + standard deviation of complete sample ; SD = social drinkers.
